# Not Just Another Crystal Field Software

**DOI:** 10.1002/jcc.70063

**Published:** 2025-03-03

**Authors:** Letizia Fiorucci, Enrico Ravera

**Affiliations:** ^1^ Department of Chemistry “Ugo Schiff” Università degli Studi di Firenze Florence Italy; ^2^ Magnetic Resonance Center Università degli Studi di Firenze Florence Italy; ^3^ Consorzio Interuniversitario Risonanze Magnetiche di Metalloproteine Florence Italy; ^4^ Max‐Planck‐Institut für Kohlenforschung Kaiser‐Wilhelm‐Platz 1 Mülheim an der Ruhr Germany; ^5^ Florence Center For Data Science Università degli Studi di Firenze Florence Italy

**Keywords:** crystal field Hamiltonian, magnetic properties, magnetometry, molecular magnetism, point charges model, single ion magnets

## Abstract

This manuscript presents NJA‐CFS, a Python‐based comprehensive toolkit for crystal field/ligand field calculations. NJA‐CFS is designed to perform simulations of electronic structure properties, including the magnetic ones, for transition metals and lanthanoid complexes, giving access to several CF/LF parametrization schemes, from point‐charge model and AOM to AILFT parameters, putting great effort in the implementation of routines for CF parameters manipulation and rotation. NJA‐CFS was designed to meet the needs of both first‐time users of crystal field theory and those who require a high degree of flexibility in the choice of crystal field parameters formalisms. In this manuscript, we present the theoretical foundations of the program routines and the comparison of NJA‐CFS calculation results either to experimental data or ab initio computations, proving the advantages that access to multiple CFPs formalism can bring in. We also present intuitive applications of the NJA‐CFS routines to didactically valuable examples, like the projection of CF/LF splitting on real d‐ and f‐ orbitals and the calculations of Tanabe–Sugano diagrams for arbitrary symmetries and with the inclusion of spin‐orbit coupling.

## Graphical abstract

NJA‐CFS is a Python toolkit for crystal field/ligand field calculations on transition metal and lanthanide complexes. It offers various parametrization schemes and tools for CF parameter manipulation and rotation. This manuscript details the theoretical foundations, compares results with experiments and ab initio calculations, and provides intuitive applications, such as orbital projections and Tanabe–Sugano diagrams.

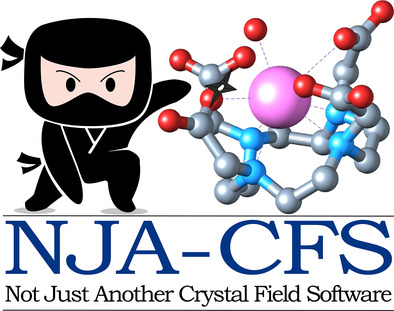

**Abbreviations**
AILFT: ab initio ligand field theory
AOM: angular overlap model
CF: crystal field
CFP: crystal field parameters
cfp: coefficients of fractional parentage
CTM: cantilever torque magnetometry
ESO: extended Stevens operator
LF: ligand field
PCM: point charge model
RME: reduced matrix elements
STO: spherical tensor operator
TTO: tesseral tensor operator

## 1 Why Another Crystal Field Software

The application of crystal‐field (CF) or ligand‐field (LF) physical Hamiltonians is a well‐established computational practice for the rationalization of optical and magnetic properties of metal complexes. Given the wide applicability and low computational cost of the CF/LF approximation, coupled with the development of several crystal‐field parametrization schemes over the years, a variety of CF/LF programs have emerged. These programs cater to diverse needs, addressing different types of metal complexes and offering specialized functionalities. Some relevant examples are: CAMMAG [1], AOMX [2], BonnMag [3], SIMPRE [4, 5, 6], CONDON [7, 8], f_electron (available at link: https://github.com/octoYot/f_electron), and PyCrystalField [9]. Not surprisingly, also projection of ab initio results onto ligand field parameters has gained significant popularity in the last decade [10, 11].

In this relatively well‐populated context, we introduce the Python suite NJA‐CFS (Not Just Another‐Crystal Field Software) with the scope of providing a comprehensive collection of functions that allow for (a) manipulating crystal field parameters, (b) navigating through the most used CF/LF parametrization formalisms, and (c) calculating the observables of interest in magnetochemistry through relatively simple scripting. NJA‐CFS is designed to handle $d^{n}$ configurations, with n from 1 to 9, and $f^{n}$ configurations, with n from 1 to 13, in both the complete basis set and a reduced microstate basis set. The flexibility and modularity of this Python program allow for easy integration of routines to compare CF/LF physical Hamiltonian results with ab initio calculations and experimental observables.

In this manuscript, we present the application of NJA‐CFS to the characterization of transition metal and lanthanoid complexes, comparing the results with first‐principle calculations and experimental data.

In addition to our core objectives, we explored the possibility of leveraging the code for educational applications. Therefore, we put great emphasis on the readability of the code, in the possibility of visualizing all the quantities that are calculated, but also in guiding the interested user through our implementation with detailed documentation and comments in the code.

## 2 Theoretical Background

In a CF/LF calculation, the main contributions included in the physical Hamiltonian are: The interelectronic repulsion term (${\hat{H}}_{\text{e‐e}}$), the spin‐orbit coupling (${\hat{H}}_{\text{s‐o}}$), the crystal field splitting (${\hat{H}}_{\text{CF}}$) and—if the system is subjected to the action of an external magnetic field—the term describing the Zeeman interaction (${\hat{H}}_{\text{Z}}$):

(1) $$\hat{H}={\hat{H}}_{\text{e–e}}+{\hat{H}}_{\text{s–o}}+{\hat{H}}_{\text{CF}}+{\hat{H}}_{\text{Z}}$$

The relative magnitude of the different terms on the splitting of the free ion states depends on the considered metal ion and the coordination environment. For transition metals, the energy contribution due to interaction with the ligands is generally larger than the spin‐orbit coupling contribution, while the opposite holds true for lanthanoid ions. This influences the number and classification scheme of the microstates (basis set) included in the CF calculation. For example, in the case of $f^{n}$ configurations the basis set can be safely reduced to include only the ground state J‐multiplet, which will be almost independent of the coordination environment. This approximation is commonly employed in CF codes, for example, SIMPRE, to reduce computational cost. However, NJA‐CFS can handle the full basis set, ensuring accurate treatment of intermediate coupling and J‐mixing. This enables a case‐by‐case assessment of the validity of the reduced basis set approximation.

An extremely convenient way to compute the Hamiltonian integrals implies the application of the theory of angular momenta in Racah's tensor operator formalism [12, 13, 14, 15, 16, 17] through the Wigner–Eckart theorem. For a generic tensor operator of degree k and order q, $T_{q}^{\left(k\right)}$, the integral computation can be broken into

(2) $$\left\langle j^{\prime }m^{\prime }|T_{q}^{\left(k\right)}|jm\rangle =\langle jmkq|j^{\prime }m^{\prime }\rangle \langle j^{\prime }\|T^{\left(k\right)}\|j\right\rangle$$

where the factors on the r.h.s. are respectively: the Clebsch‐Gordan coefficients $\left\langle jmkq|j^{\prime }m^{\prime }\right\rangle$ and the so‐called Reduced Matrix Elements (RME) $\left\langle j^{\prime }\|T^{\left(k\right)}\|j\right\rangle$. Both these factors can be found tabulated in literature, however, given the high number of states involved and in the absence of corresponding implemented versions, they can all be computed using recursive equations from a common set of coefficients (see Appendix A). To this end, the basis wave‐vectors are combined to obtain a proper wavefunction through coefficients called coefficients of fractional parentage (cfps) $G_{n,K}^{n-1,K^{\prime }}$

(3) $$|\psi (l^{n},K)\rangle =\sum_{K^{\prime }}|\psi (l^{n-1},K^{\prime })\rangle G_{n,K}^{n-1,K^{\prime }}$$

In Equation (3), the sum runs over the basis functions of the n−1 states, categorized by unique combinations of quantum numbers K. The Koster–Nielson classification scheme [18] is employed to organize these states. In fact, while the canonical $\left|L,S,J,M_{J}\right\rangle$ quantum numbers provide a fundamental description of the microstate, additional quantum numbers derived from group theory are necessary to fully characterize the states, particularly for $f^{n}$ configurations. These additional quantum numbers, devoid of direct physical interpretation, are used to classify states within a given 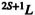 term (see Supporting Information Section S1). In the chosen representation, these additional quantum numbers are implicitly represented by a progressive numbering scheme (1, 2, 3, …) for each 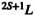 term. In this way, the composition of the wavefunctions in terms of free ion terms is straightforward and the computational burden related to the construction and computation of integrals in the determinantal product of states is avoided.

The computation of the cfps can also be performed using almost recursive equations for both $d^{n}$ and $f^{n}$ configurations, as described by Racah in [15] and by Nielson in [19], however, the procedure is not straightforward in the case of $f^{n}$ configurations for n>2 and the results are not univocal, hence adjustments based on the current formalisms are required.

In NJA‐CFS, both Clebsh–Gordan coefficients and RME are recursively computed, using tables of cfps as starting point, but the routines described in [19] for the cfps computation for $f^{n}$ configurations are also implemented and available in the program for the sake of completeness.

Within the theoretical framework outlined above, Equation (1) is written in terms of Racah's tensor operators [20], with the crystal field expressed as a distribution of discrete charges, as

(4) $$\begin{array}{c} \begin{array}{ll} \hat{H} & =\left(\frac{e^{2}}{4\pi \varepsilon_{0}}\right)\sum_{i=1}^{n}\sum_{j>i}^{n}\sum_{k=0}^{\infty }[r_{>}^{-(k+1)}\cdot r_{<}^{k}]\sum_{q=-k}^{+k}{\left(-1\right)}^{q}C_{-q}^{\left(k\right)}(i)C_{q}^{\left(k\right)}(j)\\ & +\hbar^{-2}\zeta \kappa {\left\{L^{\left(1\right)}⊗S^{\left(1\right)}\right\}}^{0}+\left(\frac{e^{2}}{4\pi \varepsilon_{0}}\right)\sum_{i=1}^{n}\sum_{\Delta }Z_{\Delta }\\ & \times \sum_{k=0}^{\infty }[r_{>}^{-(k+1)}\cdot r_{<}^{k}]\sum_{q=-k}^{+k}{\left(-1\right)}^{q}C_{-q}^{\left(k\right)}(\Delta )C_{q}^{\left(k\right)}(i)+\mu_{B}\hbar^{-1}\\ & \times \sum_{q=-1}^{+1}{\left(-1\right)}^{q}B_{-q}^{\left(1\right)}(\kappa L_{q}^{\left(1\right)}+g_{e}S_{q}^{\left(1\right)}) \end{array} \end{array}$$

where e is the electron charge, ζ is the spin‐orbit coupling constant (λ=±ζ/(2S)), κ is the orbital reduction factor, $Z_{\Delta }$ is the charge of the Δ‐donor defined as fraction of electronic charge and, $r_{>}$ and $r_{<}$ are the farthest and closest positions of electron/ligand charges respectively, with respect to the center of the reference frame, assumed coincident with the position of the metal center. $C_{q}^{\left(k\right)}$ are the tensor operators representation of the complex spherical harmonics, also called Racah normalized spherical harmonics [13]:

(5) $$C_{q}^{\left(k\right)}=\sqrt{\frac{4\pi }{2k+1}}Y_{k}^{q}$$

$L^{\left(1\right)}$ and $S^{\left(1\right)}$ are the spherical tensor representations of orbital angular momentum and spin angular momentum operators respectively and $B_{q}^{\left(1\right)}$ is the spherical tensor representation of the magnetic field vector.

The parameters used to compute each interaction term are derived either experimentally or through ab initio calculations (see Appendix A). For the electron‐electron interaction, radial integrals are parametrized using Slater–Condon parameters ($F^{k}$) or, equivalently, Racah parameters (A, B, and C). The spin‐orbit interaction term relies on the parameters ζ and κ, which also appear in the Zeeman interaction. The interaction with surrounding ligands is described either through the crystal field or ligand field approach. In the crystal field framework, the parameters represent purely electrostatic interactions, such as those derived from a point‐charge model of the ligands (PCM). By contrast, the ligand field approach incorporates the covalent aspects of the ligand‐metal interaction, as exemplified by the Angular Overlap Model (AOM).

In NJA‐CFS, both purely crystal field and ligand field approaches are incorporated. Therefore, the labels “CF” and “LF” will be used alternately in the discussion, contingent upon the specific nature of the coefficients. The term crystal field will also be broadly used to indicate the generic splitting contribution to the free ion states caused by the coordination environment.

### 2.1 Parameters From Ab Initio Calculations

An effective way for the derivation of parameters suitable for the computation of energy levels and magnetic properties in the LF/CF physical Hamiltonian framework foresees the application of an ab initio based ligand field theory (AILFT) [21, 22], available in the ORCA software for quantum chemical calculations (see Methods section). This approach allows the connection between the first‐principle CASSCF (Complete Active Space Self Consistent Field) method, possibly with the diagonal energies corrected by N‐electron valence perturbation theory (NEVPT2), and the ligand field theory.

This is performed by invoking the definition of an active space as big as the ligand‐field matrix, hence 2l+1, with l=2 for $d^{n}$ configurations and l=3 for $f^{n}$ configurations, and solving the CASSCF problem requiring the composition of the orbitals in the active space being mainly composed by the d or f metal orbitals. From this procedure, it is possible to unambiguously compute the one‐electron ligand field matrix elements ($V^{\text{LF}}$), ζ, B, and C.

Specific routines in NJA‐CFS are dedicated to the extraction and manipulation of AILFT parameters from CASSCF(/NEVPT2) ORCA calculations.

## 3 Features of NJA‐CFS

### 3.1 Crystal Field Manipulations

In the following section, the CF/LF Hamiltonian contribution, main focus of the NJA‐CFS implementation, is described in detail.

#### 3.1.1 Crystal Field Formalisms

The perturbation of the electron cloud of a metal ion, subjected to the influence of its coordination environment, can be described by a crystal‐field Hamiltonian of the type:

(6) $${\hat{H}}_{\text{CF}}=-e\overset{n}{\underset{i=1}{\sum }}V(r_{i})$$

where e is the elementary charge, the summation i runs over the n electrons in the open‐shell orbitals and $V(r_{i})$ is the potential sensed by the electron at the position $r_{i}$.

In the presence of a charge distribution ρ(R), the purely electrostatic crystal‐field potential can be expressed as the volume integral

(7) $$V(r_{i})=\int_{V}\frac{\rho (R)}{\left|R-r_{i}\right|}d\tau$$

where R indicates a general point in the ligand framework.

The integration over a continuous ρ(R) is often approximated with a discrete distribution of charges—associated with each donor—and represented by a point‐charge model (PCM). Equation (7) is thus expressed as:

(8) $$V(r_{i})=\sum_{\Delta }\frac{{\left(-\;Ze\right)}_{\Delta }}{\left|R_{\Delta }-r_{i}\right|}$$

where Z is the fraction of negative charge relative to the Δth ligand.

The potential in Equation (7) can be expanded as follows:

(9) $$\frac{1}{\left|R-r_{i}\right|}=\overset{\infty }{\underset{k=0}{\sum }}\frac{r_{<}^{k}}{r_{>}^{k+1}}P_{k}(\cos\omega )$$

where $r_{<}$ and $r_{>}$ are the shortest and longest distances respectively and $P_{k}(\cos\omega )$ are the Legendre polynomials relative to the angle ω between the two position vectors. Generally, the ligands are more distant than the electron of the metal ion itself, therefore Equation (9) becomes:

(10) $$\frac{1}{\left|R-r_{i}\right|}=\overset{\infty }{\underset{k=0}{\sum }}\frac{r_{i}^{k}}{R^{k+1}}P_{k}(\cos\omega )$$

$P_{k}(\cos\omega )$ can be further expanded using the spherical harmonics addition theorem [23]. According to that, ω can be decomposed in the polar angles: (θ,ϕ), which describe the distribution of ligand charges, and $\left(\theta_{i},\phi_{i}\right)$, which describe the position of the ith electron:

(11) $$P_{k}(\cos\omega )=\sqrt{\frac{4\pi }{2k+1}}\overset{k}{\underset{q=-k}{\sum }}Y_{k}^{q*}(\theta ,\phi )Y_{k}^{q}(\theta_{i},\phi_{i})$$

leading to the final expression:

(12) $$V(r_{i})=\overset{\infty }{\underset{k=0}{\sum }}\sqrt{\frac{4\pi }{2k+1}}\left[\overset{k}{\underset{q=-k}{\sum }}Y_{k}^{q}(\theta_{i},\phi_{i})\int_{V}\rho (R)\frac{r_{i}^{k}}{R^{k+1}}Y_{k}^{q*}(\theta ,\phi )d\tau \right]$$

which in the PCM reads:

(13) $$V(r_{i})=\overset{\infty }{\underset{k=0}{\sum }}\sqrt{\frac{4\pi }{2k+1}}\left[\overset{k}{\underset{q=-k}{\sum }}Y_{k}^{q}(\theta_{i},\phi_{i})\underset{\Delta }{\sum }-Z_{\Delta }e\frac{r^{k}}{R_{\Delta }^{k+1}}Y_{k}^{q*}(\theta_{\Delta },\phi_{\Delta })\right]$$

The radial part of this expression and the angular part describing the ligand distribution are usually factorized in the crystal field parameters (CFPs), with general expression [24]:

(14) $$V(r_{i})=\underset{kq}{\sum }c_{kq}(R)Y_{k}^{q}(\theta_{i},\phi_{i})$$

The summation over k is generally reduced to values 2 and 4 for $d^{n}$ and 2, 4, and 6 for $f^{n}$ configurations.

**FIGURE 1:**
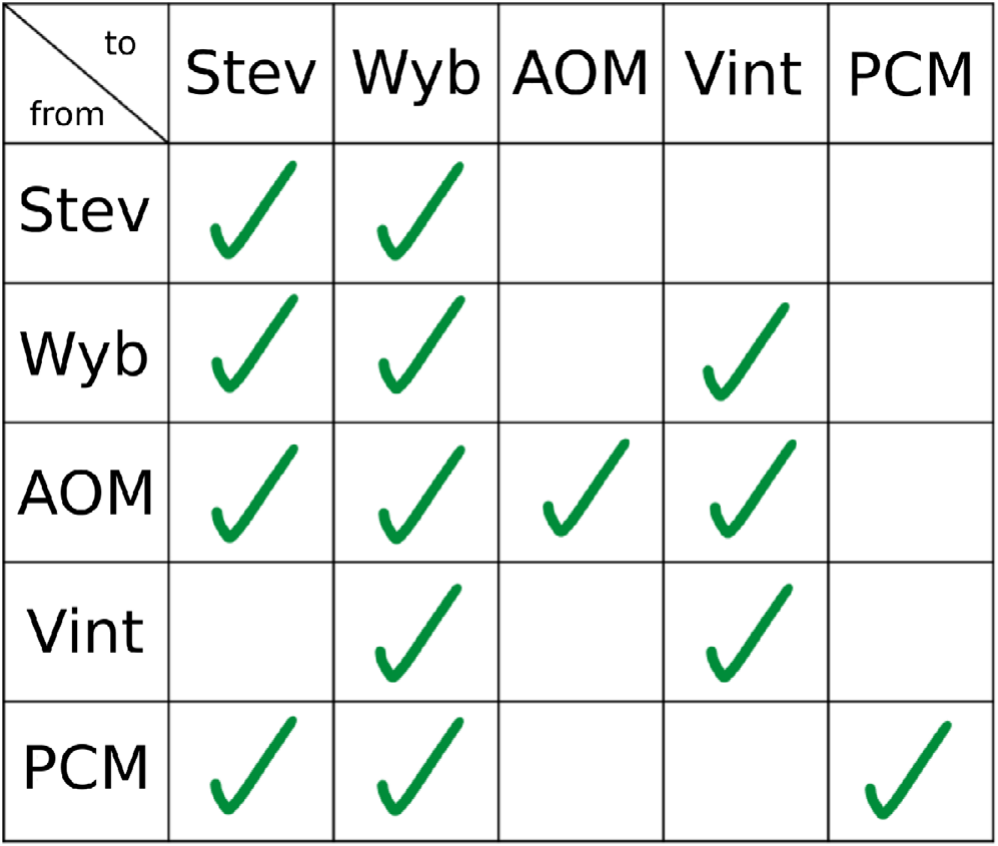
Scheme of CFPs conversion options for NJA‐CFS. The elements on diagonal cells represent the type of coefficients that can be fed into the program. The elements out of the diagonal represent the possible single or multi‐step conversions that can be performed among the CFPs formalism. **Stev**: Extended Stevens operators, **Wyb**: Wybourne convention, **AOM**: Angular Overlap Model, **Vint**: One‐electron ligand field matrix elements, PCM*: Point Charge Model. *The point charge model is not a proper formalism of crystal‐field coefficients, however, it is a formalism for the crystal field parametrization, therefore it has been included.

This factorization can be performed in many ways, giving rise to the multitudes of formalism that are available in literature. We implemented the most common—and their conversion factors—in NJA‐CFS (see Figure 1).

Stevens (1952) was the first to show that the matrix elements of the spherical harmonics in Equation (14), within the same angular momentum manifold, could be calculated by replacing the spherical harmonics by operator equivalents having similar transformation properties [25]. Since then, tensor operators (TOs) of angular momenta have been widely used in the construction of crystal field Hamiltonians [26, 27]. Two classes of TOs can be identified: the spherical tensor operators (STOs) and the tesseral tensor operators (TTOs), which are the operator equivalents to the spherical (complex) and tesseral (real) harmonics respectively. The conventional Stevens operators (CSOs) and the extended Stevens operators (ESOs) belong to the TTO class. Both of them are generally indicated as $B_{k}^{q}$, where q≥0 for the CSOs and −k≤q≤k for the ESOs. The first are not used anymore since they do not transform consistently under a general rotation of coordinates for some crystal symmetries [28]. Nowadays, the most used operator equivalents are the ESOs [29]. According to this approach, the crystal field Hamiltonian in a single $J(f^{n})$‐multiplet (or $L(d^{n})$‐multiplet) can be expressed as:

(15) $${\hat{H}}_{\text{CF}}^{\text{Stevens}}=\underset{k,q}{\sum }B_{k}^{q}O_{k}^{q}(J\;\text{or}\;L)$$

where $O_{k}^{q}$ are the ESOs, either defined in terms of total angular momentum J or orbital angular momentum L.

Another widespread notation used to express the crystal field Hamiltonian, generally used for $f^{n}$ multiplets, in terms of ESOs is the following:

(16) $${\hat{H}}_{\text{CF}}^{\text{Stevens}}=\sum_{k,q}A_{k}^{q}\langle r^{k}\rangle \theta_{k}O_{k}^{q}$$

where $A_{k}^{q}$ are the CFPs (like the $B_{k}^{q}$), $\left\langle r^{k}\right\rangle$ are the expectation value of $r^{k}$ (available in literature [30, 31]) and the $\theta_{k}$ are the multiplicative Stevens factors, indicated as α, β, and γ in the original article by Stevens, for the rank k=2,4,6 respectively (tabulated in [25]).

On the other side, an example of the application of the STOs formalism is represented by the Wybourne parametrization scheme.

According to Wybourne [32], the crystal field Hamiltonian can be defined as:

(17) $${\hat{H}}_{\text{CF}}^{\text{Wybourne}}=\underset{k,q,i}{\sum }B_{q}^{k}C_{q}^{\left(k\right)}(\theta_{i},\phi_{i})$$

where the sum over i runs on the electrons in the chosen (open‐shell) configuration and −k≤q≤k [33]. Alternatively, by rewriting Equation (11) as [34]:

(18) $$P_{k}(\cos\omega )=\frac{4\pi }{2k+1}\left[Y_{k}^{0}Y_{k}^{0}(i)+\overset{k}{\underset{q=1}{\sum }}(Y_{k}^{-q}Y_{k}^{q}(i)+Y_{k}^{q}Y_{k}^{-q}(i))\right]$$

where the spherical harmonics with (i) refer to ith‐electron position while the others refer to the position of the ligand, the Hamiltonian can be expressed in the expanded form as [35]:

(19) $$\begin{array}{c} \begin{array}{ll} & {\hat{H}}_{\text{CF}}^{\text{Wybourne}}=-e\underset{k,i}{\sum }\left[B_{0}^{k}C_{0}^{\left(k\right)}(i)+\overset{k}{\underset{q=1}{\sum }}(B_{q}^{k}(C_{-q}^{\left(k\right)}(i)+{\left(-1\right)}^{q}C_{q}^{\left(k\right)}(i))\right.\\ & \;+\left.{B^{\prime }}_{q}^{k}\text{i}(C_{-q}^{\left(k\right)}(i)-{\left(-1\right)}^{q}C_{q}^{\left(k\right)}(i)))\right]\; \end{array} \end{array}$$

The $B_{q}^{k}$ and ${B^{\prime }}_{q}^{k}$ are the real and complex part of the CFPs in this formalism respectively.

Due to the operators' properties, the Stevens CFPs $B_{k}^{q}$ (or $A_{k}^{q}$) in Equations (15) and (16) are real‐valued, whereas the Wybourne CFPs $B_{q}^{k}$ are in general complex. All operators of the STO class are simply related to each other by specific numerical coefficients arising from the Wigner–Eckhart theorem while the same is not true for the operators of the TTO class [33]. Furthermore, while Equations (15) and (16) are valid only in J(or L)‐constant manifolds, expression Equation (17) is applicable to the complete set of microstates.

In NJA‐CFS, it is possible to convert CFPs in Stevens' formalism in the Wybourne's one and to compute both of them in the CF approximation from a PCM of the coordination environment (see Appendix B).

##### 3.1.1.1 Introduction of Covalent Contribution in the Parametrization of Metal‐Ligand Interaction

The original formulation of crystal field theory relies on a purely electrostatic model to describe metal‐ligand interactions. While this approximation is suitable for lanthanoid complexes due to the minimal delocalization of f orbitals, it provides only a qualitative understanding of energy levels for transition metal ions. This limitation arises from the strong covalent character of bonding in transition metal complexes, necessitating a more sophisticated ligand field approach.

A potential approach involves utilizing one‐electron ligand field matrix elements calculated using AILFT for the computation of the corresponding CFPs (more properly called LFPs). This method provides a molecular orbital‐based description of the metal complex.

As demonstrated in the previous section, the ligand‐field potential can be expressed as a linear combination of spherical harmonics multiplied by coefficients. This is true also at one‐electron level:

(20) $$\left\langle l,m_{l}|V|l,m_{l}^{\prime }\rangle =\sum_{\Delta }c_{kq}^{\Delta }\langle l,m_{l}|Y_{k}^{q}|l,m_{l}^{\prime }\right\rangle$$

where Δ runs over the donor atoms and $\left\langle l,m_{l}|V|l,m_{l}^{\prime }\right\rangle$ are one‐electron ligand field integrals in the complex basis, elements of the one‐electron ligand field matrix ($V^{\text{LF}}$). Since the $\left\langle l,m_{l}|Y_{k}^{q}|l,m_{l}^{\prime }\right\rangle$ integrals can be easily solved using the tensor operator formalism, what we get is a series of equations that express the $c_{kq}^{\Delta }$ coefficients, that is, the LFPs, in terms of $\left\langle l,m_{l}|V|l,m_{l}^{\prime }\right\rangle$ [36, 37]. Conveniently, this can be expressed in matrix form as:

(21) c=CM

where c and M are vectors containing the (l+1)(2l+1) conversion coefficients and $V^{\text{LF}}$ matrix elements respectively, and C is the (l+1)(2l+1)×(l+1)(2l+1) coefficient matrix. This implementation allows easily also the reverse process, that is, compute the ligand field matrix elements from the CFPs via $M=C^{-1}c$. Further details on this conversion are reported in the SI.

Another alternative to the (electrostatically‐based) crystal field models, is the angular overlap model (AOM), implemented also in other numerous LF software (e.g., CAMMAG, AOMX, and BonnMag). Analogously to the other ligand‐field treatment, also this one is based on one‐electron operators. It assumes that the antibonding‐orbital energies ($E^{*}$) are determined by covalent perturbation weak enough to be proportional to the square of appropriate overlap integrals:

(22) $$E^{*}={\left(A_{t}^{d}\right)}^{2}e_{t}$$

where d indicates the metal d orbital and t the symmetry of the ligand orbital (i.e., σ,π…). $A_{t}^{d}$ is an angular factor expressing the fact that the chosen global coordinate frame of the metal atom is not generally coincident with the local frame to which the symmetry of the ligand functions are referred. $e_{t}$ is the angular overlap parameter enclosing the information on the overlap integral between the metal and ligand orbitals and it is different for each ligand type and for each bonding mode. Contributes arising from the different ligands to this antibonding energy are considered additive. A general expression for the one‐electron matrix elements within the AOM model is the following:

(23) $$\left\langle l,u|V|l,v\rangle =\sum_{\Delta }\sum_{t}^{\text{modes}}D_{ut}^{l}(\Delta )D_{vt}^{l}(\Delta )e_{t}(\Delta \right)$$

where $D_{ut}^{l}$ and $D_{vt}^{l}$ are the matrix elements of the transformation of the real metal orbitals into each ligand coordinate frame. In direction cosines, the D matrix elements for the modes $e_{\sigma }$, $e_{\pi_{c}}$ and $e_{\pi_{s}}$ can be expressed for l=2 with Equation (9) of [36] and for l=3 with coefficients in Table 1 of [37].

Based on this model, it is possible to derive an additional way to compute the CFPs. Using Equation (23) one can compute the $V^{\text{LF}}$ matrix, and from there one can proceed with Equation (21).

#### 3.1.2 Rotations

Since the (complex) crystal field coefficients in the Wybourne convention can be expressed in terms of linear combinations of spherical harmonics, they maintain the same transformation properties. Consequently, an arbitrary rotation of the CFPs can be performed using the Wigner D‐matrix (D). In NJA‐CFS we provided two different options to do so.

One way is to define the rotation based on the intrinsic Z‐Y‐Z Euler angles convention as:

(24) $$D_{q^{\prime }q}^{k}(\alpha ,\beta ,\gamma )=e^{-\text{i}q^{\prime }\alpha }d_{q^{\prime }q}^{k}(\beta )e^{-\text{i}q\gamma }$$

where k is equal to 2l (l=2 for $d^{n}$ configurations and l=3 for $f^{n}$ configurations), q runs from −k to k and $d_{q^{\prime }q}^{k}(\beta )$ is the element of the Wigner's (small) d‐matrix, defined as:

(25) $$\begin{array}{c} \begin{array}{ll} & d_{q^{\prime }q}^{k}(\beta )={\left[(k+q^{\prime })!(k-q^{\prime })!(k+q)!(k-q)!\right]}^{1/2}\\ & \;\times \overset{s_{\max}}{\underset{s=s_{\min}}{\sum }}\left[\frac{{\left(-1\right)}^{q^{\prime }+q+s}{\left(\cos\frac{\beta }{2}\right)}^{2k+q-q^{\prime }-2s}{\left(\sin\frac{\beta }{2}\right)}^{q^{\prime }-q+2s}}{(k+q-s)!s!(q^{\prime }-q+s)!(k-q^{\prime }-s)!}\right]\; \end{array} \end{array}$$

where the sum over s runs over such values that the factorials are non‐negative, that is, $s_{\min}=\max(0,q-q^{\prime })$ and $s_{\max}=\min(k+q,k-q^{\prime })$.

However, Euler angles suffer from—besides the ambiguity among the different conventions [38], —the gimbal‐lock problem (i.e., the indetermination of one angle for particular values of the other two), which severely accurse their applications for numerical optimization. Therefore, we provide the Wigner rotation matrices in quaternions, which was implemented following Lynden‐Bell and Stone procedure in [39] and briefly described in Appendix C.

Alternatively, the rotation can be performed on the (2k+1)×(2k+1) one‐electron ligand field matrix. However, since the elements of $V^{\text{LF}}$ are real‐valued (contrary to the Wybourne CFPs) while the elements of $D^{k}$ are complex, the rotation matrix is transformed using:

(26) $$U=\left(\begin{array}{ccccc} 1/\sqrt{2} & 0 & 0 & 0 & -\text{i}/\sqrt{2}\\ 0 & -1/\sqrt{2} & 0 & \text{i}/\sqrt{2} & 0\\ 0 & 0 & 1 & 0 & 0\\ 0 & 1/\sqrt{2} & 0 & \text{i}/\sqrt{2} & 0\\ 1/\sqrt{2} & 0 & 0 & 0 & \text{i}/\sqrt{2} \end{array}\right)$$

for k=2 (for $d^{n}$ configurations), and:

(27) $$U=\left(\begin{array}{ccccccc} -1/\sqrt{2} & 0 & 0 & 0 & 0 & 0 & \text{i}/\sqrt{2}\\ 0 & 1/\sqrt{2} & 0 & 0 & 0 & -\text{i}/\sqrt{2} & 0\\ 0 & 0 & -1/\sqrt{2} & 0 & \text{i}/\sqrt{2} & 0 & 0\\ 0 & 0 & 0 & 1 & 0 & 0 & 0\\ 0 & 0 & 1/\sqrt{2} & 0 & \text{i}/\sqrt{2} & 0 & 0\\ 0 & 1/\sqrt{2} & 0 & 0 & 0 & \text{i}/\sqrt{2} & 0\\ 1/\sqrt{2} & 0 & 0 & 0 & 0 & 0 & \text{i}/\sqrt{2} \end{array}\right)$$

for k=3 (for $f^{n}$ configurations), as:

(28) $$D^{k}=U^{†}D^{k}U$$

At this point, each element of $V^{\text{LF}}$ can be converted, for an arbitrary rotation R either defined in terms of Euler angles or quaternions, as follows:

(29) $$\begin{array}{c} V_{MM^{\prime }}^{\text{LF},\text{rot},k}=\underset{m^{\prime }}{\sum }\underset{m^{\prime \prime }}{\sum }[D_{Mm^{\prime }}^{k}(R)][D_{M^{\prime }m^{\prime \prime }}^{k}{\left(R\right)}^{*}]V_{m^{\prime }m^{\prime \prime }}^{\text{LF},k} \end{array}$$

for M, $m^{\prime }$, $M^{\prime }$, and $m^{\prime \prime }$ in range −k,…,k.

##### 3.1.2.1 Wavefunction Optimization

The crystal field orientation affects the wavefunction composition but not the energy levels. The orientation in which the wavefunctions are the most compact corresponds to the principal axes system of the crystal field.

An optimization of this kind which involves only the ground state wavefunction, performed on a grid of Euler angles, is available in SIMPRE [4]. We propose the same idea in NJA‐CFS, adapted to the computational features of a Python software.

In NJA, the optimization can be performed either on a grid of REPULSION angles [40] or with simulated annealing minimization algorithm [41]. The CFPs are rotated using the functions described in the previous section.

**FIGURE 2:**
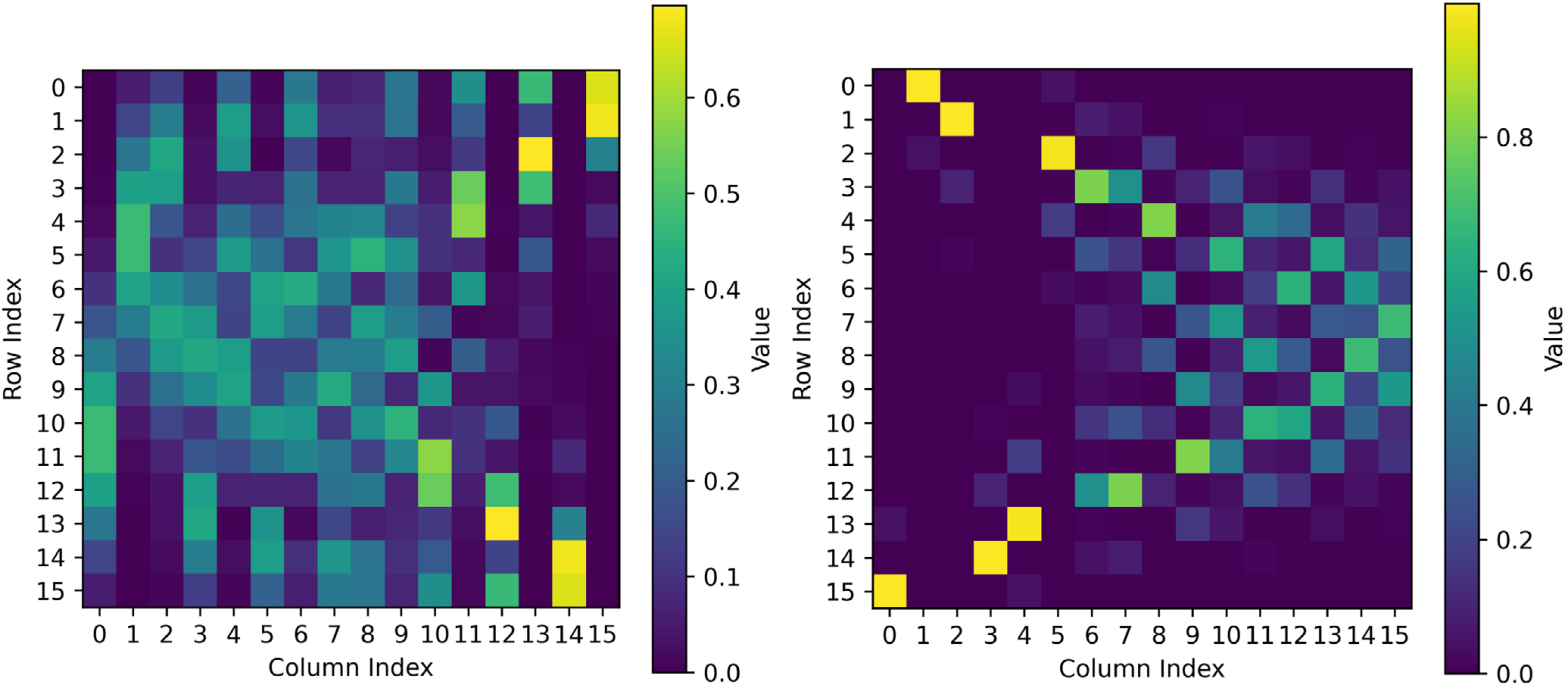
Eigenfunctions composition expressed by the squared module of the wavefunction components obtained from the Hamiltonian diagonalization, before (left) and after (right) optimization of the CFPs orientation for the Dybbpn complex performed through a fitting procedure. The CFPs were computed from a PCM of the metal coordination environment. The PCM is reported in Table S7 and the code in listing S1.

We applied this procedure to the optimization of the orientation of a PCM representing the Dybbpn complex [42]. Figure 2 shows the eigenfunctions composition before and after the optimization implemented in NJA‐CFS. It is intuitive that the susceptibility tensor (see Section 3.3) computed after the optimization would be more diagonal compared to the same tensor computed without the orientation adjustment. This is caused by the fact that, in general, the reduction of the out‐of‐diagonal terms in the Hamiltonian matrix also produces a decrease in the mixed terms in the magnetic properties.

### 3.2 Energy Levels Diagrams

The simulation starts with the definition of a *calculation()* object, that takes as input the configuration of choice. The calculations can be performed on transition metal complexes, for configurations from $d^{1}$ to $d^{9}$ (from 10, for $d^{1}$, to 252, for $d^{5}$, microstates in the complete basis), and on lanthanoid complexes, for configurations from $f^{1}$ to $f^{13}$ (from 14, for $f^{1}$, to 3432, for $f^{7}$, microstates in the complete basis).

At this stage, the basis set is generated. In NJA‐CFS, the calculation can be performed either on the entire population of microstates or on a subset of states, which can help in facing the inevitable increase of computational time that computations on $f^{n}$ configuration can cause (it can be applied also to $d^{n}$ configurations). The basis set reduction can be performed at multiple levels. The drastic way is, in case of $f^{n}$ configurations (for n from 2 to 12), to use only the ground J multiple. In many cases, this results in almost identical energy level splittings with respect to the complete calculation (see Table 1), in fact, this procedure is often implemented in CF software (e.g., SIMPRE). Alternatively, it is possible to select a specific number of free ion terms based on their spin multiplicity, to mimic what one would do in a CAS calculation in ORCA (see Methods section).

**TABLE 1:** The energy level values (in ${\text{cm}}^{-1}$) relative to the ground state multiplet for [DyDOTA(H_2_O)]^−^ ($C_{4}$) complex obtained from the complete approach (top row) and the ground‐only calculation, including only the ^6^H_15/2_ (bottom row). All the states are doubly degenerate.

Complete basis	7.181	63.027	95.133	145.614	209.124	288.136	386.291
^6^H_15/2_ basis	7.501	63.598	96.586	148.052	212.781	293.036	392.296

The computation of energy levels and eigenfunctions composition is performed using the *MatrixH()* function, where the parameters used in the calculations of the Hamiltonian integrals are also defined. As anticipated in Section 2, the electron‐electron interaction requires the Slater–Condon parameters $F^{2}$ and $F^{4}$ for $d^{n}$ configurations and $F^{2}$, $F^{4}$, and $F^{6}$ for $f^{n}$ configurations (that can be also computed starting from the Racah's A, B, and C parameters). For spin‐orbit coupling, the SO coupling coefficient (ζ) and the orbital reduction factor (κ) have to be defined. Concerning the crystal‐field contribution, the starting point can be any of the formalisms presented in the previous section. Lastly, the Zeeman term requires the definition of a magnetic field vector and the orbital reduction factor κ. The $F^{k}$ and ζ parameters can be directly read in tables available inside the NJA program or read (together with the $V^{\text{LF}}$) from an AILFT ORCA output.

Once the calculation of energy levels and wavefunctions terminates, the energy levels splitting diagram (see Figure S1 and listing S2) can be interactively open in a *matplotlib* interface and the state composition in terms of |LSJ⟩ and/or $\left|LSJM_{J}\right\rangle$ can be displayed.

The way the Hamiltonian is computed does not require any prior knowledge of the relative magnitude of the terms in Equation (1). This means that the program allows the inspection of the relative magnitude of the different terms for any metal complex in the allowed configurations. For example, the comparison of Figures S1 and S2 clearly demonstrates why the orbital contribution to the magnetic moment of a lanthanoid complex tends to be strongly dequenched by the spin‐orbit contribution, while the CF quenching is much heavier in the transition metal complexes (see listing S2 and S3).

#### 3.2.1 Tanabe–Sugano Diagrams

A nice application of these functions is the construction of the Tanabe–Sugano diagrams, found in any introductory inorganic chemistry textbook for metal complexes with the most common coordination geometries, for example, $O_{h}$ and $T_{d}$ symmetries. A more exhaustive collection of these diagrams can be found in dedicated manuscripts [17], however, their consultation is not straightforward and they are not valid for a non‐symmetric complex.

These diagrams are constructed by computing the energy levels including only the electron‐electron interaction contribution and the crystal field contribution.

In NJA‐CFS, they can be obtained using the script listed in SI (listing S4). In Figure S3 we show the Tanabe–Sugano diagrams for a $d^{7}$ configuration for different symmetries: $O_{h}$, $C_{4v}$, $D_{3h}$, and $T_{d}$ (see Table S1–S4).

One could also include the spin‐orbit contribution in the Hamiltonian, and see how the diagrams change under the effect of the ζ value across, for example, $d^{8}$ group in square planar complexes (see Figure S4).

#### 3.2.2 Orbital Splitting Diagrams

The interpretation of the effects of the crystal‐field splitting based on the effect that this contribution has on the energy levels splitting diagram is not necessarily intuitive. We are often used to learn about CF/LF effect based on the splitting that it induces on the d or f metal orbitals.

It could become useful for this reason to project the energy levels splitting pattern onto the d or f orbitals, which is obtained by diagonalizing and plotting the eigenvalues of the $V^{\text{LF}}$ matrix computed from a set of $B_{q}^{k}$ (see Figure S5, Tables S5 and S6, and listing S5).

### 3.3 Magnetic Properties

Functions for the study of the field, temperature, and orientation dependence of the magnetic properties of the metal complexes are included in dedicated NJA‐CFS routines. The computation of magnetic properties starts with the calculation of the energy levels, followed by the definition of a *Magnetics()* object.

The available routines are:

- *effGval()*, for the computation of the effective g matrix for a Kramers doublet of states |i⟩ and |j⟩, using the following definition:
(30) $$g_{kl}=\langle i|\mu_{k}|i\rangle \langle i|\mu_{l}|i\rangle +2\langle i|\mu_{k}|j\rangle \langle j|\mu_{l}|i\rangle +\langle j|\mu_{k}|j\rangle \langle j|\mu_{l}|j\rangle$$
  with: $\mu_{k}=\kappa L_{k}+g_{e}S_{k}$ (k,l=x,y,z) and the square root of the double of the eigenvalues of this matrix are the actual effective g values for the x, y and z directions,
- *susceptibility*_*field()*, for the computation of the magnetization and magnetic susceptibility scalar fields, computed as
(31) $$\begin{array}{ll} & \;\;\;M(B)=\frac{\mu_{B}}{3}\frac{\sum_{k}\sum_{i}\mu_{k}e^{-\frac{E{\left(B\right)}_{i}}{k_{B}T}}}{\sum_{i}e^{-\frac{E{\left(B\right)}_{i}}{k_{B}T}}}\; \end{array}$$
(32) $$\begin{array}{ll} & \chi (B)=\mu_{0}\mu_{B}\frac{M(B+\delta B)-M(B-\delta B)}{2\delta B}\; \end{array}$$
  where $E{\left(B\right)}_{i}$ is the energy value for the level i computed for the magnetic field vector B, $k_{B}$ is the Boltzmann constant, T is the temperature and k=x,y,z (see listing S6). The calculation is performed for each magnetic field vector B defined on the surface of a sphere according to a set of angles determined either with the REPULSION algorithm [40] or with the golden spiral method. Such fields can be plotted with a dedicated function.
- *susceptibility*_*B*_*ord1()*, for the computation of the magnetization vector and susceptibility tensor, defined, for a certain magnetic field vector $B=[B_{x},B_{y},B_{z}]$, as:
(33) $$\begin{array}{ll} & M_{k}=\mu_{B}\frac{\sum_{i}{\left(\mu_{k}\right)}_{i}e^{-\frac{E_{i}}{k_{B}T}}}{\sum_{i}e^{-\frac{E_{i}}{k_{B}T}}}\; \end{array}$$
(34) $$\begin{array}{ll} & \;\chi_{kl}=\mu_{B}\mu_{0}\frac{\partial M_{k}}{\partial B_{l}}\; \end{array}$$
  The numerical derivative is computed according to the Ridders' method of polynomial extrapolation, which also returns an estimation of the derivative error. This algorithm was adapted from the function reported at p. 182 of [43] (see SI for the implementation).

**FIGURE 3:**
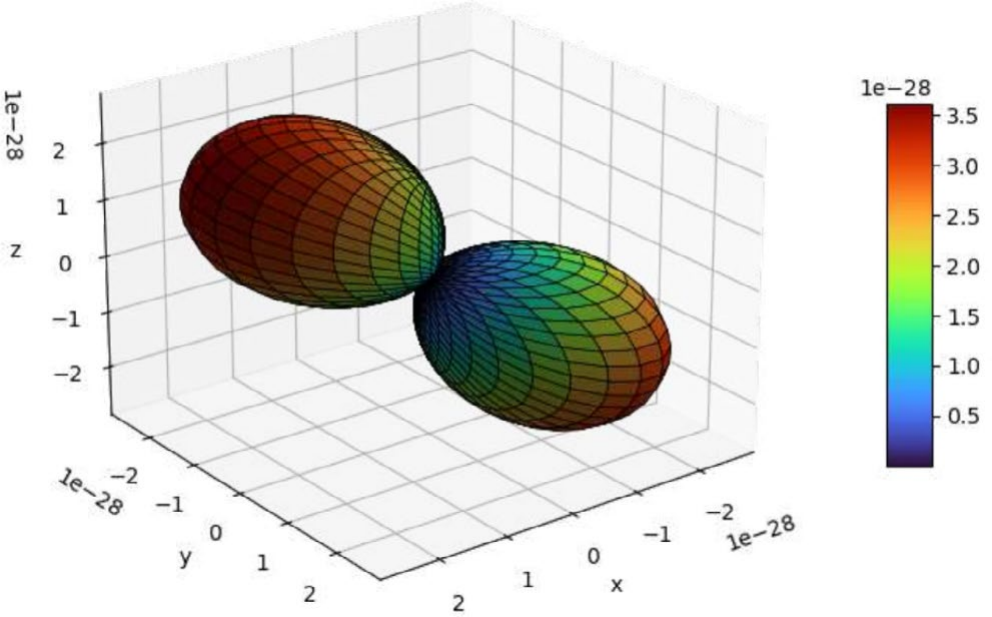
Representation of the anisotropy of the susceptibility tensor for the DyDOTA complex (with $B_{k}^{q}$ from [44] rotated from the effective g reference frame to the $ab^{\prime }c*$ reference frame of the crystal). The code listing for the generation of this figure is reported in the SI (see listing S7).

The tensorial properties can be represented as their projection over a grid of points (see Figure 3).

#### 3.3.1 Simulation of Magnetic Torque for CTM

Cantilever Torque Magnetometry (CTM) is a classic technique for the characterization of paramagnetic centers in crystals. CTM measures the magnetic torque (τ) exerted by a paramagnetic crystal mounted on a cantilever and immersed in a magnetic field. This defines the tendency of a magnetic dipole moment to align along the direction of the applied magnetic field (B).

The magnetic torque is simply calculated as the cross product between B and the magnetic moment of the system (M):

(35) τ=M×B

If the system is composed of multiple magnetic centers, the total torque is defined by the sum of the magnetic torques of the single centers:

(36) $$\tau =\underset{i}{\sum }m_{i}\times B$$

From these general equations, closed forms that explicitly include the contributions from the magnetic susceptibility tensor components can be derived [45].

**FIGURE 4:**
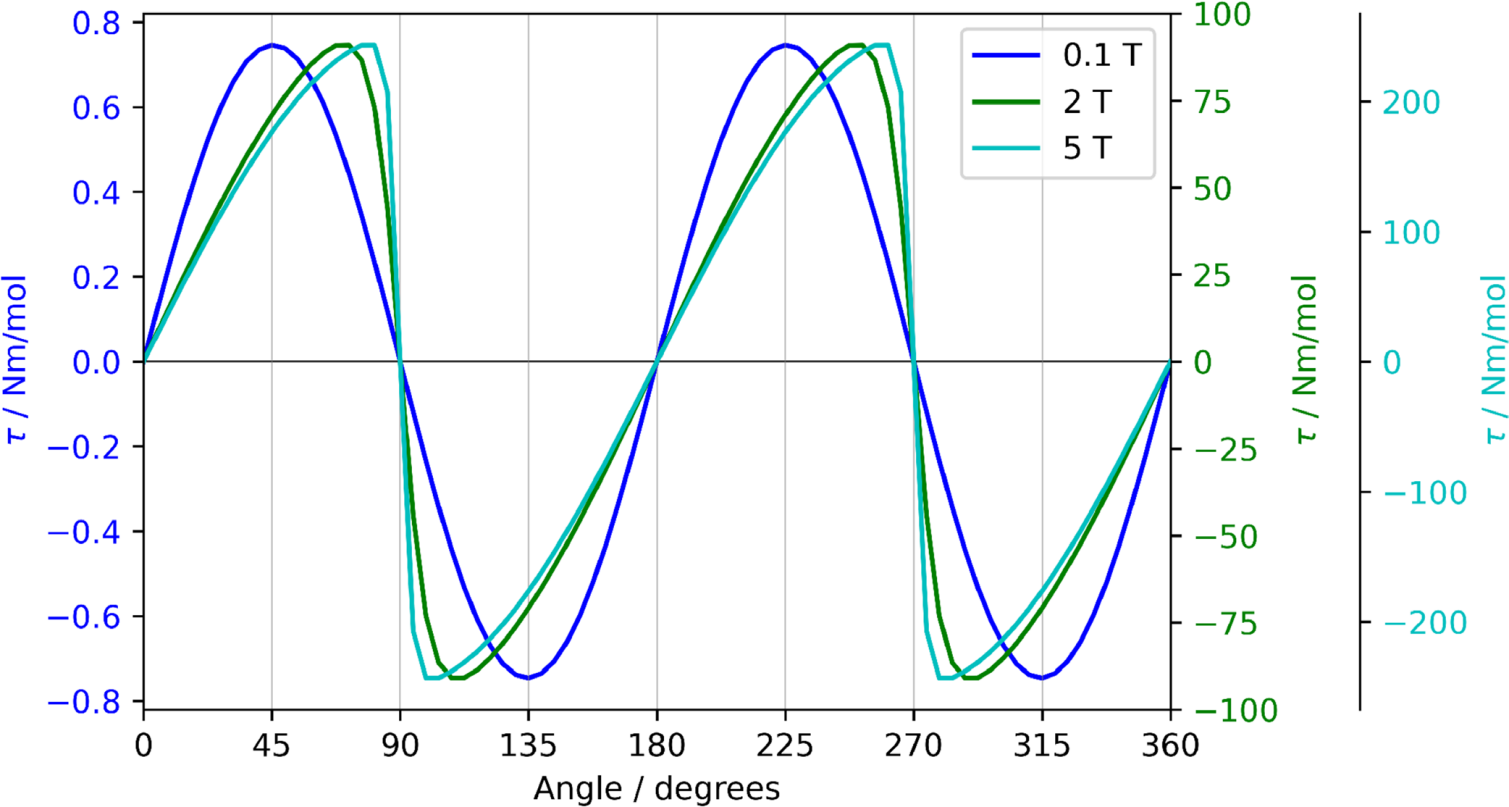
Simulation of magnetic torque for terbium(III) complex TbPc_2_ (the $A_{k}^{q}\langle r^{k}\rangle$ are taken from [46]) for 0.1 T, 2 T, and 5 T at 2 K. The code listing for the generation of this figure is reported in the SI (see listing S8).

The implementation of such equations leads to profiles of the type in Figure 4, for the TbPc_2_ (Pc = dianion of phthalocyanine) complex.

### 3.4 Comparison With Ab Initio Results

Given the goal of CF/LF programs as a rapid approximation to ab initio calculations, the validation of results obtained from NJA‐CFS must be carried out through comparison with ab initio results. This necessity arises because ab initio methods, despite being computationally intensive, are grounded in first principles and provide highly precise results.

**FIGURE 5:**
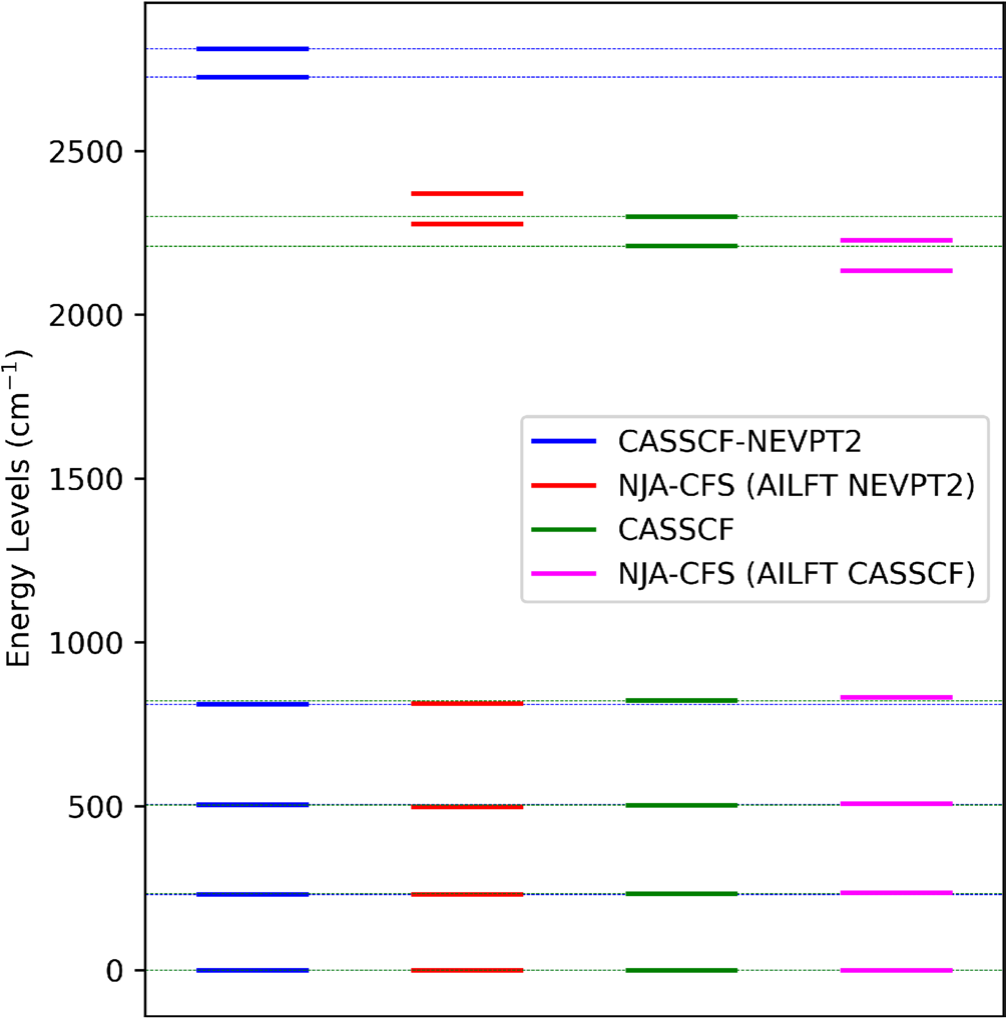
Comparison of the lowest energy levels values computed with NJA‐CFS and those computed with a QC calculation for the cobalt(II) $d^{7}$ complex CoTp_2_. The parameters used in NJA‐CFS were obtained from the ab initio LF treatment implemented in ORCA, including all possible microstates (i.e., 10 quartets and 40 doublets) in the CASSCF(7,5) calculation either with or without the NEVPT2 correction. Also in the NJA‐CFS calculation, all microstates were used.

**FIGURE 6:**
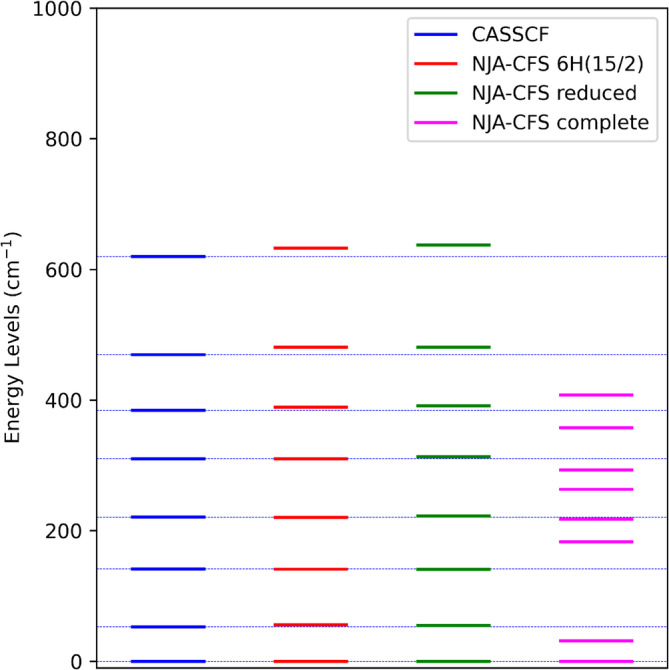
Comparison of the 16 lowest energy levels values computed with NJA‐CFS for different bases and those computed with a QC calculation for the dysprosium(III) $f^{9}$ complex DyDOTA. The parameters used in NJA‐CFS were obtained from the ab initio LF treatment implemented in ORCA, including all 21 sextuplets in the CASSCF(9,7) calculation. The label *16H(15/2)* refers to the NJA‐CFS calculation performed on the ground state J‐multiplet only, the term *reduced* refers to a basis set reduced to the 21 sextuplets and, lastly, the term *complete* refers to the calculation performed including the complete basis.

Figures 5 and 6 show for example the comparison between the energy levels computed from ab initio calculations and those from the NJA program employing the AILFT parameters. An important note when performing these tests is that, in order to congruently compare ab initio and NJA‐CFS results, the basis set has to be reduced to the same basis set used in the ab initio calculation (see listing S9). For example, in the DyDOTA calculation, only the 21 lowest sextets were included in the active space and hence the same was done also in the NJA‐CFS replica. The inclusion of all states in the NJA calculation worsens the result. From these figures, it is also evident that the extremely good agreement for the $f^{n}$ system that we get using the proper basis and the qualitative good agreement for the cobalt(II) complex. This sustains an already well‐known and intuitive observation, that is, the CF/LF approximation is generally more suitable for lanthanoid complexes with respect to $d^{n}$ systems. Additionally, Figure 5 reveals that the inclusion of dynamic correlation with NEVPT2 is not totally parametrized by the AILFT fitting, hence the best agreement is obtained at the CASSCF level. However, this would not affect the susceptibility tensor computation, since the contribution of the magnetic moment expectation value is weighted by the Boltzmann factor.

The validation proceeded with the comparison of the susceptibility tensors computed at zero magnetic field and room temperature for the DyDOTA complex and its temperature dependence (see Figure S6).

**FIGURE 7:**
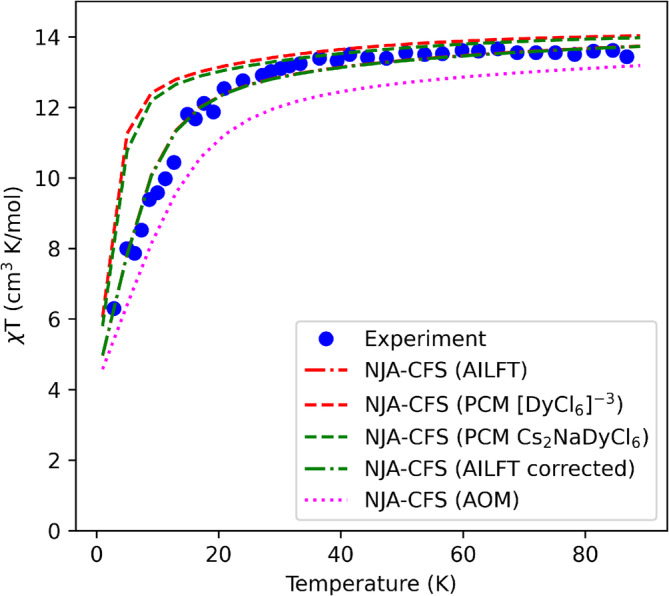
Magnetic susceptibility temperature dependence of Cs_2_NaDyCl_6_ elpasolite complex computed with NJA‐CFS using different sources of CFPs, compared to the experimental data from [47]. AILFT = $F^{k}$, ζ and $V^{\text{LF}}$ from CASSCF(9,7) on [DyCl_6_]^−3^ model. PCM [DyCl_6_]^−3^=*B*_*q*_^*k*^ computed for the charges distribution in table S8, and $F^{k}$, ζ from AILFT. PCM Cs_2_NaDyCl_6_=*B*_*q*_^*k*^ computed for the charges distribution including positive charges in the model and replicating the unit cell twice for each lattice vector, and $F^{k}$, ζ from AILFT. AILFT corrected = $F^{k}$, ζ and $V^{\text{LF}}$ from CASSCF(9,7) on [DyCl_6_]^−3^ model and an additional contribution to the $V^{\text{LF}}$ from the PCM of the Cs_2_NaDyCl_6_ structure without the central [DyCl_6_]^−3^ unit. AOM = $e_{\sigma }$, $e_{\pi }$, $F_{k}$ and ζ from Table 4 in [3].

Finally, we applied different crystal field parametrization schemes for the simulation of the experimental susceptibility temperature dependence of the Cs_2_NaDyCl_6_ elpasolite crystal [47], that is, (1) AILFT for the simplified representation of the crystal [DyCl_6_]^3−^ and published on the CASSCF tutorial guide from ORCA (reported in listing S10), (2) the $B_{q}^{k}$ computed for the PCM of [DyCl_6_]^−3^ (see Table S8), (3) the $B_{q}^{k}$ computed for a larger PCM of the Cs_2_NaDyCl_6_ crystal (reported in a separate xyz file as SI) with all the charges of the lattice, that is, +1 for Cs, +1 for Na, +3 for Dy and −1 for Cl, (4) AILFT corrected by the PCM of approach n. 3, and (5) the AOM parameters from literature [48, 49] and reproduced in [3] (see Figure 7). This comparison shows that the experimental data are best reproduced using the AILFT parameters from protocol n. 1. The PCM model performs well for temperatures between 40 and 80 K (protocol n. 2) and the correction for the complete crystal model produces an improvement in the agreement (protocol n. 3), while the same correction applied to the AILFT $V^{\text{LF}}$ matrix in protocol n. 4 does not change the situation significantly (the lines referring to “NJA‐CFS (AILFT)” and “NJA‐CFS (AILFT corrected)” appear perfectly superimposed). The use of the AOM parameters in protocol n. 5 leads to an underestimation of the susceptibility for the selected temperature range. The energy levels for the ground multiplet are collected in Table 2.

**TABLE 2:** The energy level values (in ${\text{cm}}^{-1}$) of the ground multiplet ^6^H_15/2_ for the Cs_2_NaDyCl_6_ elpasolite crystal, are presented computed according to the protocols in Figure 7. The NJA calculations for AILFT, PCM [DyCl_6_]^−3^, PCM Cs_2_NaDyCl_6_, and AILFT corrected were performed using the 21 sextets as basis, while for the AOM calculations we used the minimum number of states to get equivalent energy values to those reproduced in Table S2 of [3], that is, 11 sextets and 13 quartets. The code is available in listing S10.

n. state	AILFT	PCM [DyCl_6_]^−3^	PCM Cs_2_NaDyCl_6_	AILFT corrected	AOM
1–2	0.0	0.0	0.0	0.0	0.0
3–4	17.745	6.880	7.653	17.285	28.985
5–6	17.849	6.880	8.637	18.734	28.985
7–8	82.068	54.422	64.798	84.572	176.546
9–10	167.961	82.887	98.001	170.010	305.537
11–12	168.038	82.887	98.984	171.601	305.537
13–14	204.726	104.517	122.591	206.743	379.605
15–16	204.780	104.517	126.798	210.888	379.605

This represents a nice example of how NJA is able to compare results from different CFPs sources and even allows the correction of such CFPs through the combinations of different formalisms. To the best of our knowledge, this is not possible in any other implementation.

## 4 Conclusions

We have presented NJA‐CFS, a comprehensive CF/LF toolbox based on Python scripting. Its functions can be used to compute energy levels, wavefunctions, as well as the magnetic properties (magnetic susceptibility tensor, effective g‐tensor, magnetization and susceptibility scalar fields, magnetic torque), for transition metals and lanthanoid complexes. In the development, we have put great emphasis on the readability of the code and in the conversion among different formalisms. To prove the usability of this collection, we provided example scripts that encompass several applications, including the comparison with ab initio results and experimental data, and education‐oriented cases.

## 5 Methods

The program is available at: https://github.com/letiziafiorucci/NJA‐CFS.

More details about computational requirements and usage guidelines are reported in the documentation.

NJA‐CFS is organized into two Python scripts: nja_cfs_vX.py houses all program functions, neatly organized in classes. The script test_nja.py provides a suite of examples and test functions to ensure the program's correctness and reliability. Supporting materials, including tables and documentation outlining computational requirements and usage guidelines, are kept in dedicated folders.

The code was tested in Python version 3.12 on Ubuntu 22.04 LTS inside a dedicated Anaconda environment, however, it is also compatible with previous versions of Python 3 and other OS. The additional mandatory dependencies and their versions are: numpy (version 2.0.2) [50], matplotlib (version 3.9.2) [51] and scipy (version 1.14.1) [52]. The program could also work with different versions of the above‐mentioned packages. Additional optional dependencies include: numba (version 0.60.0) [53] and sympy (version 1.13.3).

For the ab initio calculations, the ORCA software v6 was used [54]. The AILFT mode was activated by imposing *actorb = dorbs* or *forbs* in the *CAS* module [21, 55]. The correction for dynamic correlation (NEVPT2) [56, 57] was applied in the 3d transition metal ion cases. The second‐order Douglas‐Kroll‐Hess Hamiltonian [58] was employed to account for the scalar relativistic effects, analogously to what was done in [59]. The DKH‐def2‐TZVP basis was used for all atoms except for the lanthanoid ions, which were treated with the SARC2‐DKH‐QZVP basis [60].

For the construction of Tanabe–Sugano diagrams of the $d^{8}$ series, we employed the AILFT from CASSCF(8,5) and the DKH‐def2‐TZVP for nickel(II), and SARC‐DKH‐TZVP for palladium(II) and platinum(II). The complex structure for Ni is reported in Table S9.

### 5.1 Notes on Computational Time

We are aware of the limits of Python implementation in terms of computational time. To have an idea: The computation of energy levels, wavefunctions, and wavefunction projections for a $f^{9}$ configuration (equivalent to the $f^{5}$) in the complete basis, that is, 2002 microstates, takes between 10 and 20 min on an average laptop, depending on the processor performances. The computational time increases even more, non‐linearly, for $f^{6}$ and $f^{7}$ configurations and equivalents. For this reason, we put great effort into options for basis set reduction. Additionally, in the GitHub directory is available a NJA‐CFS version which employs the numba package to improve the computational efficiency of magnetic properties computation, nja_cfs_red.py. Thanks to the use of numba the computational time is more than halved. However, this adaptation is not available at the moment for the computation of energy levels and will be added in future updates.

## Supporting information




**Data S1.** Additional supporting information may be found in the online version of the article at the publisher’s website.

**Data S1.** Supplementary Code Data.

## Data Availability

The data that support the findings of this study are openly available in NJA‐CFS at https://github.com/letiziafiorucci/NJA‐CFS.

## Appendix A Equations for Integrals Evaluation

### A.1. Electron‐Electron Interaction

The matrix elements for electron‐electron interaction are diagonal in J and $M_{J}$ [20]:

(A1)
$$\begin{array}{c} \begin{array}{l} \langle l^{n}\nu LSJM_{J}|{\hat{H}}_{e-e}|l^{n}\nu^{\prime }L^{\prime }S^{\prime }J^{\prime }M_{J}^{\prime }\rangle =\langle l^{n}\nu LS\|{\hat{H}}_{e-e}\|l^{n}\nu^{\prime }L^{\prime }S^{\prime }\rangle \delta_{J,J^{\prime }}\delta_{M_{J}^{\prime },M_{J}^{\prime }}\; \end{array} \end{array}$$

where the seniority quantum number ν is made explicit among the possible (α) and:

(A2)

with k=0,2,4 for $d^{n}$ configurations and k=0,2,4,6 for $f^{\;n}$ configurations.

For a two‐electron configuration the equation is simplified in:

(A3)
$$\begin{array}{c} \begin{array}{l} \langle l^{2}\nu LS\|{\hat{H}}_{e-e}\|l^{2}\nu^{\prime }L^{\prime }S^{\prime }\rangle =\delta_{L,L^{\prime }}\delta_{S,S^{\prime }}\overset{\infty }{\underset{k=0}{\sum }}F_{ll}^{k}{\left[\langle l\|C^{\left(k\right)}\|l\rangle \right]}^{2}{\left(-1\right)}^{L}\left\{\begin{array}{ccc} l & l & k\\ l & l & L \end{array}\right\}\; \end{array} \end{array}$$

where

(A4)
$$F_{ll}^{k}=\int \int \frac{r_{<}^{k}}{r_{>}^{k+1}}R_{l}^{2}(r_{1})R_{l}^{2}(r_{2})r_{1}^{2}r_{2}^{2}dr_{1}dr_{2}$$

and the reduced matrix elements of the Racah operator are defined as:

(A5)
$$\langle l\|C^{\left(k\right)}\|l\rangle ={\left(-1\right)}^{l}{\left[(2l+1)(1l+1)\right]}^{1/2}\left(\begin{array}{ccc} l & k & l\\ 0 & 0 & 0 \end{array}\right)$$

The 3‐j symbol $\left(\begin{array}{ccc} a & b & c\\ A & B & C \end{array}\right)$ represents the coupling of two angular momenta and can be computed using the Racah's closed form [13]:

(A6)
$$\begin{array}{l} \begin{array}{ll} \left(\begin{array}{ccc} a & b & c\\ A & B & C \end{array}\right)= & {\left(-1\right)}^{a-b-C}\left[\frac{(a+b-c)!(b+c-a)!(c+a-b)!}{(a+b+c+1)!}\right]\\ & \times {\left[(a+A)!(a-A)!(b+B)!(b-B)!(c+C)!(c-C)!\right]}^{1/2}\\ & \times \overset{n_{\max}}{\underset{n=n_{\min}}{\sum }}{\left(-1\right)}^{n}[n!(c-b+n+A)!(c-a+n-B)!(a+b-c-n)!\\ & \times (a-n-A)!{\left(b-n+B)!\right]}^{-1}\; \end{array} \end{array}$$

with:

$$\begin{array}{c} \begin{array}{ll} n_{\min} & =\max\{0;-c+b-A;-c+a+B\}\\ n_{\max} & =\min\{a+b-c;b+B;a-A\} \end{array} \end{array}$$

For n>2 and l=2, the equation is the following:

(A7)
$$\begin{array}{c} \left\langle l^{n}\nu LS\|{\hat{H}}_{e-e}\|l^{n}\nu^{\prime }L^{\prime }S^{\prime }\rangle =\delta_{L,L^{\prime }}\delta_{S,S^{\prime }}\underset{k=0,2,4}{\sum }F_{ll}^{k}\cdot c^{k}(l^{n}\nu \nu^{\prime }LS\right) \end{array}$$

with the angular coefficients, for k>0:

(A8)
$$\begin{array}{c} \begin{array}{ll} c^{k}(l^{n}\nu \nu^{\prime }LS)\; & =\frac{1}{2}{\left\langle l\|C^{\left(k\right)}\|l\right\rangle }^{2}\\ & \times \;\left\{\frac{1}{2L+1}\underset{\nu^{\prime \prime }L^{\prime \prime }}{\sum }\langle l^{n}\nu LS\|U^{\left(k\right)}\|l^{n}\nu^{\prime \prime }L^{\prime \prime }S\rangle \right.\\ & \left.\times \;\langle l^{n}\nu^{\prime }LS\|U^{\left(k\right)}\|l^{n}\nu^{\prime \prime }L^{\prime \prime }S\rangle -\frac{n}{2l+1}\delta_{\nu ,\nu^{\prime }}\right\}\; \end{array} \end{array}$$

and for k=0:

(A9)
$$c^{0}(l^{n}\nu \nu^{\prime }LS)=\frac{n(n-1)}{2}\delta_{\nu ,\nu^{\prime }}$$

The integrals computation for n>2 and l=3 is more complex [15, 32]. Equation (A7) is usually rewritten as:

(A10)
$$\begin{array}{c} \langle l^{n}\nu LS\|{\hat{H}}_{e-e}\|l^{n}\nu^{\prime }LS\prime =\underset{k=0,2,4,6}{\sum }f_{k}F^{k}=\underset{k=0,2,4,6}{\sum }f^{\;k}F_{k} \end{array}$$

with the $F^{k}$ defined as Equation (A19), and $f^{k}=D_{k}f_{k}$, with $D_{k}$ are those that relate $F^{k}$ to $F_{k}$.

Although the operator $\left(C_{1}^{\left(k\right)}\cdot C_{2}^{\left(k\right)}\right)$ in Equation (A2) is scalar with respect to $R_{3}$, it does not have the transformation properties of the group $R_{7}$ and $G_{2}$, and therefore we have to use linear combinations of such operators. The matrix elements are re‐defined as:

(A11)
$${\hat{H}}_{\text{e}-\text{e}}=\underset{k=0,1,2,3}{\sum }e_{k}E^{k}$$

where $e_{k}$ are related to the $f^{k}$ by the expressions:

(A12)
$$\begin{array}{c} e_{0}=f^{0}=n(n-1)\\ e_{1}=\frac{9}{7}f^{0}+\frac{1}{42}f^{2}+\frac{1}{77}f^{4}+\frac{1}{462}f^{6}\\ e_{2}=\frac{143}{42}f^{2}-\frac{130}{77}f^{4}+\frac{35}{462}f^{6}\\ e_{3}=\frac{11}{42}f^{2}+\frac{4}{77}f^{4}-\frac{7}{462}f^{6} \end{array}$$

whereas the $E^{k}\text{s}$ are linear combinations of the $F_{k}\text{s}$:

(A13)
$$\begin{array}{ll} E^{0} & =F_{0}-10F_{2}-33F_{4}-286F_{6}\\ E^{1} & \;=\frac{70F_{2}+231F_{4}+2002F_{6}}{9}\\ E^{2} & \;=\frac{F_{2}-3F_{4}+7F_{6}}{9}\\ E^{3} & \;=\frac{5F_{2}+6F_{4}-91F_{6}}{3}\; \end{array}$$

The $e_{i}\text{s}$ can be computed with the following procedure:

(A14)
$$\begin{array}{ll} e_{0} & =n(n-1)/2\; \end{array}$$

(A15)
$$\begin{array}{ll} e_{1}(f^{n}USL) & =9(n-v)/2+v(v+1)/4-S(S+1)\; \end{array}$$

(A16)
$$\begin{array}{c} e_{2}(f^{n}USLU^{\prime }SL)=\pm e_{2}(WUL,WU^{\prime }L)\\ \;=\pm \sum_{\gamma }x_{\gamma }(W,UU^{\prime })(U|\chi_{\gamma }|U^{\prime }) \end{array}$$

(A17)
$$\begin{array}{c} \begin{array}{ll} e_{3}(\;f^{n}vUSLv^{\prime }U^{\prime }SL) & =y(f^{n},vSU,v^{\prime }SU^{\prime })(U|\phi (L)|U^{\prime })-\Omega \delta_{UU^{\prime },vv^{\prime }}\; \end{array} \end{array}$$

where all the factors (and additional closed forms) necessary for the computation of $e_{2}$ and $e_{3}$ for all $f^{n}$ configurations are available in [15]. NJA reads these factors from a table, however, the routine for their calculation is available in the software.

The parameters that have to be passed to the program for the computation of this contribution are the Slater–Condon parameters $F^{k}$. The parameter $F^{0}$ can be omitted since we are interested in the splitting of the terms, not in their absolute position on the energy scale.

Care must be taken for the superscript k, since:

(A18)
$$\begin{array}{ll} F^{0} & =F_{0}\\ F^{2} & =49F_{2}\\ F^{4} & =441F_{4}\; \end{array}$$

for $d^{n}$ and:

(A19)
$$\begin{array}{c} F^{0}=F_{0}\\ F^{2}=225F_{2}\\ F^{4}=1089F_{4}\\ F^{6}=184,041F_{6}/25 \end{array}$$

for $f^{n}$.

### A.2. Spin‐Orbit Coupling

The spin‐orbit coupling contribution is diagonal in J and $M_{J}$

(A20)
$$\begin{array}{l} \langle l^{n}\nu LSJM_{J}|{\hat{H}}_{so}|l^{n}\nu^{\prime }L^{\prime }S^{\prime }J^{\prime }M_{J}^{\prime }\rangle =\kappa \langle l^{n}\nu LS\|{\hat{H}}_{so}\|l^{n}\nu^{\prime }L^{\prime }S^{\prime }\rangle \delta_{J,J^{\prime }}\delta_{M_{J}^{\prime },M_{J}^{\prime }}\; \end{array}$$

This reduced matrix element can be computed as:

(A21)
$$\begin{array}{c} \begin{array}{ll} & \langle l^{n}\nu LS\|{\hat{H}}_{so}\|l^{n}\nu^{\prime }L^{\prime }S^{\prime }\rangle =\zeta {\left(-1\right)}^{J+L+S^{\prime }}{\left[l(l+1)(2l+1)\right]}^{1/2}\\ & \;\;\times \left\{\begin{array}{ccc} L & L^{\prime } & 1\\ S^{\prime } & S & J \end{array}\right\}\langle l^{n}\nu LS\|V^{\left(11\right)}\|l^{n}\nu^{\prime }L^{\prime }S^{\prime }\rangle \; \end{array} \end{array}$$

where the RMEs $\left\langle l^{n}\nu LS\|V^{\left(11\right)}\|l^{n}\nu^{\prime }L^{\prime }S^{\prime }\right\rangle$ are computed according to Equation (A31).

In this case the parameters to be defined are the spin‐orbit coupling parameter ζ and the orbital reduction factor κ.

### A.3. Crystal‐Field Splitting

In this case, even if different kinds of parameters can be fed into the program (see Section 3.1.1), the formalism used for the integral resolution is the Wybourne one [32]. The crystal‐field integrals in the Racah's tensor operator formalism, according to Equation (19), can be expressed as:

(A22)
$$\begin{array}{c} \begin{array}{ll} & \left\langle \alpha SLJM_{J}|{\hat{H}}_{cf}|\alpha^{\prime }S^{\prime }L^{\prime }J^{\prime }M_{J}^{\prime }\right\rangle \\ & \;=\langle \alpha SLJM_{J}|-e\underset{i,k}{\sum }\left[B_{0}^{k}C_{0}^{\left(k\right)}(i)\right.\\ & \;\left.+\;\overset{k}{\underset{q=1}{\sum }}(B_{q}^{k}(C_{-q}^{\left(k\right)}(i)+{\left(-1\right)}^{q}C_{q}^{\left(k\right)}(i))+{B^{\prime }}_{q}^{k}\text{i}(C_{-q}^{\left(k\right)}(i)\right.\\ & \;\left.-\;{\left(-1\right)}^{q}C_{q}^{\left(k\right)}(i)))\right]|\alpha^{\prime }S^{\prime }L^{\prime }J^{\prime }M_{J}^{\prime }\rangle \; \end{array} \end{array}$$

where α represent the additional set of quantum numbers used and k has values 2 and 4 for $d^{n}$ and 2, 4, and 6 for $f^{n}$ configurations. The $B_{q}^{k}$ and ${B^{\prime }}_{q}^{k}$ are the so‐called crystal field coefficients (CFCs), that can be converted in the corresponding crystal field parameters $B_{q}^{k}$ and ${B^{\prime }}_{q}^{k}$, when solving the integral, by bringing them outside the matrix element, together with the radial parts of the wavefunctions $R_{nl}(r)$. In this way, the tensor operators are left acting solely on the angular part. The use of the same notation for both coefficients and parameters is confusing but, unfortunately, it is what is done in literature.

The CFPs are defined as:

(A23)
$$B_{0}^{k}=\int_{r=0}^{\infty }R_{nl}^{2}(r)r^{k}dr\sqrt{\frac{4\pi }{2k+1}}\underset{\Delta }{\sum }Z_{k0}^{c}(\theta_{\Delta })\frac{Z_{\Delta }e^{2}}{R_{\Delta }^{k+1}}$$

(A24)
$$B_{q}^{k}=\int_{r=0}^{\infty }R_{nl}^{2}(r)r^{k}dr\sqrt{\frac{4\pi }{2k+1}}\frac{1}{\sqrt{2}}\underset{\Delta }{\sum }Z_{kq}^{c}(\theta_{\Delta },\phi_{\Delta })\frac{Z_{\Delta }e^{2}}{R_{\Delta }^{k+1}}$$

(A25)
$${B^{\prime }}_{q}^{k}=\int_{r=0}^{\infty }R_{nl}^{2}(r)r^{k}dr\sqrt{\frac{4\pi }{2k+1}}\frac{1}{\sqrt{2}}\underset{\Delta }{\sum }Z_{kq}^{s}(\theta_{\Delta },\phi_{\Delta })\frac{Z_{\Delta }e^{2}}{R_{\Delta }^{k+1}}$$

where the sum over Δ runs over the donor atoms, represented in the point charge model. The $Z_{kq}(\theta_{\Delta },\phi_{\Delta })$ are the tesseral harmonics of degree k and order q (with q>1), defined at the polar (colatitudinal) angle $\theta_{\Delta }$ and azimuthal (longitudinal) angle $\phi_{\Delta }$ at the Δ‐th donor position. The very same equations can be expressed in terms of the (complex) spherical harmonics $Y_{k}^{q}$. $Z_{\Delta }$ and $R_{\Delta }$ are the point charge (expressed as the fraction of negative charge) and the distance from the metal ion of the Δ‐th donor respectively. For a continuous distribution of charges, an integral over the charge density can be used. The integrals over the radial part are represented by dedicated parametrizations, generally represented as $\left\langle r^{k}\right\rangle$.

The angular parts (containing the electron coordinates) of the matrix elements expressed in Equation (A22) can be calculated using the tensor operators properties, by rewriting the summation over i in terms of unit tensor $U_{q}^{k}$:

(A26)
$$\begin{array}{l} \left\langle \alpha SLJM_{J}|\underset{i}{\sum }C_{q}^{\left(k\right)}(i)|\alpha^{\prime }S^{\prime }L^{\prime }J^{\prime }M_{J}^{\prime }\right\rangle \\ \;=\langle \alpha SLJM_{J}|U_{q}^{\left(k\right)}|\alpha^{\prime }S^{\prime }L^{\prime }J^{\prime }M_{J}^{\prime }\rangle \langle l\|C^{\left(k\right)}\|l^{\prime }\rangle \; \end{array}$$

The $U_{q}^{k}$ matrix elements are diagonal in S.

Applying the Wigner–Eckart theorem, and solving the RME of $C^{\left(k\right)}$, Equation (A26) becomes:

(A27)
$$\begin{array}{l} \left\langle \alpha SLJM_{J}|\underset{i}{\sum }C_{q}^{\left(k\right)}(i)|\alpha^{\prime }SL^{\prime }J^{\prime }M_{J}^{\prime }\right\rangle \\ \;={\left(-1\right)}^{2J-M_{J}+S+L^{\prime }+k+l}{\left[(2J+1)(2J^{\prime }+1)\right]}^{1/2}\\ \;\times \;{\left[(2l+1)(2l^{\prime }+1)\right]}^{1/2}\left(\begin{array}{ccc} l & k & l^{\prime }\\ 0 & 0 & 0 \end{array}\right)\left(\begin{array}{ccc} J & k & J^{\prime }\\ -M_{J} & q & M_{J}^{\prime } \end{array}\right)\\ \;\times \;\left\{\begin{array}{ccc} J & J^{\prime } & k\\ L^{\prime } & L & S \end{array}\right\}\langle \alpha SL\|U^{\left(k\right)}\|\alpha^{\prime }SL^{\prime }\rangle \; \end{array}$$

The RMEs $\left\langle \alpha SL\|U^{\left(k\right)}\|\alpha^{\prime }SL^{\prime }\right\rangle$ can be solved using the cfps and the equations in Appendix A5.

### A.4. Zeeman Interaction

The contribution from the Zeeman interaction with the magnetic field vector is expressed as:

(A28)
$$\begin{array}{l} \left\langle l^{n}\nu LSJM_{J}|{\hat{H}}_{Z}|l^{n}\nu^{\prime }L^{\prime }S^{\prime }J^{\prime }M_{J}^{\prime }\right\rangle \\ \;=\mu_{B}\hbar^{-1}\overset{1}{\underset{q=-1}{\sum }}{\left(-1\right)}^{q}B_{-q}^{\left(1\right)}\times {\left(-1\right)}^{J-M}\left(\begin{array}{ccc} J & 1 & J^{\prime }\\ -M_{J} & q & M_{J}^{\prime } \end{array}\right)\\ \;\times \;\langle l^{n}\nu LSJ\|(\kappa L^{\left(1\right)}+g_{e}S^{\left(1\right)}\|l^{n}\nu^{\prime }L^{\prime }S^{\prime }J^{\prime }\rangle \; \end{array}$$

where the RMEs are computed as:

(A29)
$$\begin{array}{c} \begin{array}{l} \left\langle l^{n}\nu LSJ\|(\kappa L^{\left(1\right)}+g_{e}S^{\left(1\right)}\|l^{n}\nu^{\prime }L^{\prime }S^{\prime }J^{\prime }\right\rangle \\ \;=\delta_{L,L^{\prime }}\delta_{S,S^{\prime }}\hbar {\left[(2J+1)(2J^{\prime }+1)\right]}^{1/2}\\ \;\times \;\left\{k{\left[L(L+1)(2L+1)\right]}^{1/2}{\left(-1\right)}^{L+S+J+1}\left\{\begin{array}{ccc} J^{\prime } & J & 1\\ L & L & S \end{array}\right\}\right.\\ \;+\;g_{e}{\left[S(S+1)(2S+1)\right]}^{1/2}\\ \;\left.\times \;{\left(-1\right)}^{L+L^{\prime }+2S^{\prime }+J+J^{\prime }}{\left(-1\right)}^{L+S+J+1}\left\{\begin{array}{ccc} J^{\prime } & J & 1\\ S & S & L \end{array}\right\}\right\}\; \end{array} \end{array}$$

with:

$$\begin{array}{ll} B_{+1}^{\left(1\right)} & =-\frac{1}{\sqrt{2}}(B_{x}+\text{i}B_{y})\;\\ B_{-1}^{\left(1\right)} & =\frac{1}{\sqrt{2}}(B_{x}-\text{i}B_{y})\;\\ B_{0}^{\left(1\right)} & =B_{z}\; \end{array}$$

In this case the parameters needed are the magnetic field vector $B=[B_{x},B_{y},B_{z}]$ and the orbital reduction factor κ.

### A.5. Reduced Matrix Elements

The RMEs for the different tensor operators used in the equations presented in this appendix can be computed, for a generic rank k and configuration $l^{n}$, with the following expressions:

(A30)
$$\begin{array}{ll} \left(\alpha LS\|U^{\left(k\right)}\|\alpha^{\prime }L^{\prime }S^{\prime }\right) & =\delta_{SS^{\prime }}n{\left[(2L+1)(2L^{\prime }+1)\right]}^{1/2}\\ & \;\times \;\underset{\alpha^{\prime \prime },L^{\prime \prime },S^{\prime \prime }}{\sum }(l^{n-1}(\alpha^{\prime \prime }L^{\prime \prime }S^{\prime \prime })lSL|\}l^{n}\alpha LS)\\ & \;\times \;(l^{n-1}(\alpha^{\prime \prime }L^{\prime \prime }S^{\prime \prime })lSL|\}l^{n}\alpha^{\prime }L^{\prime }S^{\prime })\\ & \;\times \;{\left(-1\right)}^{K+L+l+L^{\prime \prime }}\left\{\begin{array}{ccc} l & L & L^{\prime \prime }\\ L^{\prime } & l & k \end{array}\right\}\; \end{array}$$

and:

(A31)
$$\begin{array}{l} \left(\alpha LS\|V^{\left(1k\right)}\|\alpha^{\prime }L^{\prime }S^{\prime }\right)\\ \;=\frac{3}{2}{\left[(2L+1)(2L^{\prime }+1)(2S+1)(2S^{\prime }+1)\right]}^{1/2}\\ \;\times \;\underset{\alpha^{\prime \prime },L^{\prime \prime },S^{\prime \prime }}{\sum }(l^{n-1}(\alpha^{\prime \prime }L^{\prime \prime }S^{\prime \prime })lSL|\}l^{n}\alpha LS)\\ \;\times \;(l^{n-1}(\alpha^{\prime \prime }L^{\prime \prime }S^{\prime \prime })lSL|\}l^{n}\alpha^{\prime }L^{\prime }S^{\prime })\\ \;\times \;{\left(-1\right)}^{k+L+l+L^{\prime \prime }}\left\{\begin{array}{ccc} l & L & L^{\prime \prime }\\ L^{\prime } & l & k \end{array}\right\}\\ \;\times \;{\left(-1\right)}^{1+S+1/2+S^{\prime \prime }}\left\{\begin{array}{ccc} 1/2 & S & S^{\prime \prime }\\ S^{\prime } & 1/2 & 1 \end{array}\right\}\; \end{array}$$

In both equations, the 6‐j symbol $\left\{\begin{array}{ccc} a & b & c\\ A & B & C \end{array}\right\}$ represents the coupling of three angular momenta, and can be solved using the Racah's equation [13]:

(A32)
$$\begin{array}{l} \left\{\begin{array}{ccc} a & b & c\\ A & B & C \end{array}\right\}={\left(-1\right)}^{a+b+A+B}f(abc)f(ABc)f(AbC)f(aBC)\\ \;\times \;\overset{n_{\max}}{\underset{n=n_{\min}}{\sum }}{\left(-1\right)}^{n}(a+b+A+B+1-n)!/\left[n!(a+b-c-n)!\right.\\ \;\left.\times \;(A+B-c-n)!(a+B-C-n)!(A+b-C-n)!\right.\\ \;\left.\times \;(-a-A+c+C+n)!(-b-B+c+C+n)!\right]\; \end{array}$$

with:

$$f(abc)={\left[\frac{(a+b-c)!(a-b+c)!(-a+b+c)!}{(a+b+c+1)!}\right]}^{1/2}$$

and:

$$\begin{array}{ll} n_{\min} & =\max\{0;a+A-c-C;b+B-c-C\}\;\\ n_{\max} & =\min\{a+b+A+B+1;a+b-c;A+B-c;\;\\ & \;a+B-C;A+b-C\}\; \end{array}$$

## Appendix B B.1.Cfps From PCM

The conversion coefficients from Stevens and Wybourne conventions used in this program, that is,

(B1)
$$\begin{array}{ll} \lambda_{kq} & =B_{q}^{k}/A_{k}^{q}\langle r^{k}\rangle \;\text{for}\;q\geq 0 \end{array}$$

(B2)
$$\begin{array}{ll} \lambda_{k|q|} & ={B^{\prime }}_{\left|q\right|}^{k}/A_{k}^{q}\langle r^{k}\rangle \;\text{for}\;q<0\; \end{array}$$

are listed in Table 1 of [6]. These conversion factors are ONLY valid if the phase convention in Equation (19) is used. Appropriate sign changes should be applied to such conversion coefficients if alternative conventions are used [61, 62].

The formalism used in NJA‐CFS for the computation of the CF integrals is the Wybourne one. These CFPs can either be fed into the function *MatrixH()* (that performs the computation of energy levels and eigenfunctions) directly or through the definition of a PCM model, applying the following equations [6]:

(B3)
$$\begin{array}{ll} B_{0}^{k} & =\sqrt{\frac{4\pi }{2k+1}}\langle r^{k}\rangle \underset{\Delta }{\sum }\frac{Z_{\Delta }e^{2}}{R_{\Delta }^{k+1}}Y_{k}^{0}(\theta_{\Delta },\phi_{\Delta })\; \end{array}$$

(B4)
$$\begin{array}{ll} B_{q}^{k} & =\sqrt{\frac{4\pi }{2k+1}}{\left(-1\right)}^{q}\langle r^{k}\rangle \underset{\Delta }{\sum }\frac{Z_{\Delta }e^{2}}{R_{\Delta }^{k+1}}\text{Re}Y_{k}^{q}(\theta_{\Delta },\phi_{\Delta })\; \end{array}$$

(B5)
$$\begin{array}{ll} {B^{\prime }}_{q}^{k} & =\sqrt{\frac{4\pi }{2k+1}}{\left(-1\right)}^{q}\langle r^{k}\rangle \underset{\Delta }{\sum }\frac{Z_{\Delta }e^{2}}{R_{\Delta }^{k+1}}\text{Im}Y_{k}^{q}(\theta_{\Delta },\phi_{\Delta })\; \end{array}$$

with q>0. Alternatively, the $B_{q}^{k}$ can be computed from $A_{k}^{q}\langle r^{k}\rangle$ using the $\lambda_{kq}$ factors, using the *from*_*Akqrk*_*to*_*Bkq()*. A function for the direct calculation of the Stevens coefficients is also implemented in *calc*_*Akqrk()*from PCM:

(B6)
$$\begin{array}{ll} A_{q}^{0} & =\frac{4\pi }{2k+1}\underset{\Delta }{\sum }\frac{Z_{\Delta }e^{2}}{R_{\Delta }^{k+1}}Z_{k0}(\theta_{\Delta },\phi_{\Delta })p_{k0}\; \end{array}$$

(B7)
$$\begin{array}{ll} A_{k}^{q} & =\frac{4\pi }{2k+1}\underset{\Delta }{\sum }\frac{Z_{\Delta }e^{2}}{R_{\Delta }^{k+1}}Z_{kq}^{\text{c}}(\theta_{\Delta },\phi_{\Delta })p_{kq}\; \end{array}$$

(B8)
$$\begin{array}{ll} A_{k}^{-q} & =\frac{4\pi }{2k+1}\underset{\Delta }{\sum }\frac{Z_{\Delta }e^{2}}{R_{\Delta }^{k+1}}Z_{kq}^{\text{s}}(\theta_{\Delta },\phi_{\Delta })p_{kq}\; \end{array}$$

where $p_{kq}$ are tesseral harmonics' numerical prefactors and the tesseral harmonics are defined as:

(B9)
$$\begin{array}{ll} Z_{k0} & =Y_{k}^{0}\; \end{array}$$

(B10)
$$\begin{array}{ll} Z_{kq}^{\text{c}} & =\frac{1}{\sqrt{2}}(Y_{k}^{-q}+{\left(-1\right)}^{q}Y_{k}^{q})\; \end{array}$$

(B11)
$$\begin{array}{ll} Z_{kq}^{\text{s}} & =\frac{i}{\sqrt{2}}(Y_{k}^{-q}-{\left(-1\right)}^{q}Y_{k}^{q})\; \end{array}$$

Notice the phase convention used in this context.

## Appendix C C.1.Wigner D Matrix in Quaternions

The derivation starts from the definition of the simplest Wigner D‐matrix, for k=1/2. Expressing $D^{1/2}$ as:

(C1)
$$D^{1/2}=\left(\begin{array}{cc} A & B\\ -B^{*} & A^{*} \end{array}\right)$$

where the elements are complex functions of α, β and γ, which satisfy:

(C2)
$$AA^{*}+BB^{*}=1$$

The connection between these matrix elements and the quaternion description can be found by expanding the matrix as:

(C3)
$$D^{1/2}=A_{\text{Re}}I-B_{\text{Im}}i-B_{\text{Re}}j-A_{\text{Im}}k$$

where I is the 2×2 unit matrix, $A_{\text{Re}}$ and $A_{\text{Im}}$ ($B_{\text{Re}}$ and $B_{\text{Im}}$) are the real and imaginary parts of A (B) respectively, and the 2×2 matrices i, j, k are:

(C4)
$$\begin{array}{ll} i & =\left(\begin{array}{cc} 0 & -\text{i}\\ -\text{i} & 0 \end{array}\right)\; \end{array}$$

(C5)
$$\begin{array}{ll} j & =\left(\begin{array}{cc} 0 & -\text{i}\\ \text{i} & 0 \end{array}\right)\; \end{array}$$

(C6)
$$\begin{array}{ll} k & =\left(\;\begin{array}{cc} \;-\text{i} & \;0\\ 0 & \;\text{i} \end{array}\right)\; \end{array}$$

which satisfy the multiplication rules:

(C7)
$$i^{2}=j^{2}=k^{2}=-I$$

The parameters of a normalized quaternion $\left(q_{0},q\right)$ are related to A and B as:

(C8)
$$\begin{array}{ll} A & =q_{0}-\text{i}q_{3}\; \end{array}$$

(C9)
$$\begin{array}{ll} B & =-q_{2}-\text{i}q_{1}\; \end{array}$$

Given that any rotation matrix can be built from $D^{1/2}$, since an eigenfunction of angular momentum with l=m=L (where L=k in this case) can be constructed from 2L spin =1/2 functions, the top left element of the Wigner rotation matrix in the customary arrangement (labeling the rows and columns in the order k,k−1,…,−k) is given by:

(C10)
$$D_{kk}^{k}=A^{2k}$$

The rest of the matrix can be constructed using the operators $\hat{ℜ}$ and $\hat{B}$ for the generation of the matrix elements on the right and beneath respectively:

(C11)
$$\begin{array}{ll} \hat{ℜ}D_{q^{\prime }q}^{k} & ={\left(k(k+1)-q(q+1)\right)}^{1/2}D_{q^{\prime }q-1}^{k}\; \end{array}$$

(C12)
$$\begin{array}{ll} \hat{B}D_{q^{\prime }q}^{k} & ={\left(k(k+1)-q^{\prime }(q^{\prime }+1)\right)}^{1/2}D_{q^{\prime }-1q}^{k}\; \end{array}$$

The effect of $\hat{ℜ}$ and $\hat{B}$ on A, $A^{*}$, B and $B^{*}$ can be deduced from the matrix in Equation (C1). Additionally, since both operators are differential operators, they obey to the usual rules for the differentiation of products. The following relation was also used in the matrices construction:

(C13)
$$D_{-q^{\prime }-q}^{k}={\left(-1\right)}^{q^{\prime }-q}{\left(D_{q^{\prime }q}^{k}\right)}^{*}$$

The matrices for k=2 and k=3 are reported in Tables C1 and C2.

**TABLE C1:** Wigner D matrix in quaternions, for *k* = 2 the missing elements can be derived with Equation (C13). $Z=q_{0}^{2}-q_{1}^{2}-q_{2}^{2}+q_{3}^{2}=AA^{*}-BB^{*}$.

$q^{\prime }$	q	Matrix element
2	2	$A^{4}$
2	1	$2A^{3}B$
2	0	$\sqrt{6}A^{2}B^{2}$
1	2	$-2A^{3}B^{*}$
1	1	$A^{2}(2Z-1)$
1	0	$\sqrt{6}ABZ$
0	2	$\sqrt{6}A^{2}B^{*2}$
0	1	$-\sqrt{6}AB^{*}Z$
0	0	$1/2(3Z^{2}-1)$
−1	2	$-2AB^{*3}$
−1	1	$B^{*2}(2Z+1)$
−2	2	$B^{*4}$
−2	1	$-2A^{*}B^{*3}$

**TABLE C2:** Wigner D matrix in quaternions, for *k* = 3 the missing elements can be derived with Equation (C13). $Z=q_{0}^{2}-q_{1}^{2}-q_{2}^{2}+q_{3}^{2}=AA^{*}-BB^{*}.$.

$q^{\prime }$	q	Matrix element
3	3	$A^{6}$
3	2	$\sqrt{6}BA^{5}$
3	1	$\sqrt{15}B^{2}A^{4}$
3	2	$2\sqrt{5}B^{3}A^{3}$
2	3	$-\sqrt{6}A^{5}B^{*}$
2	2	$A^{4}(3Z-2)$
2	1	$\left(1/2)\sqrt{10}BA^{3}(3Z-1\right)$
2	0	$\sqrt{30}B^{2}A^{2}Z$
1	3	$\sqrt{15}A^{4}B^{*2}$
1	2	$\left(1/2)\sqrt{10}A^{3}B^{*}(1-3Z\right)$
1	1	$\left(1/4)A^{2}(15Z^{2}-10Z-1\right)$
1	0	$\left(1/2)\sqrt{3}AB(5Z^{2}-1\right)$
0	3	$-2\sqrt{5}A^{3}B^{*3}$
0	2	$\sqrt{30}A^{2}B^{*2}Z$
0	1	$\left(1/2)\sqrt{3}AB^{*}(1-5Z^{2}\right)$
0	0	$\left(1/2)(5Z^{3}-3Z\right)$
−1	3	$\sqrt{15}A^{2}B^{*4}$
−1	2	$-(1/2)\sqrt{10}AB^{*3}(1+3Z)$
−1	1	$\left(1/4)B^{*2}(15Z^{2}+10Z-1\right)$
−2	3	$-\sqrt{6}AB^{*5}$
−2	2	$B^{*4}(3Z+2)$
−2	1	$-(1/2)\sqrt{10}A^{*}B^{*3}(3Z+1)$
−3	3	$B^{*6}$
−3	2	$-\sqrt{6}A^{*}B^{*5}$
−3	1	$\sqrt{15}B^{*4}A^{*2}$

In order to apply this matrix to the different orders of CFPs, the real‐valued Wigner coefficients are organized in a complex array ($B^{k}$):

(C14)
$$B^{k}=[B_{k}^{k},B_{k-1}^{k},\text{…},B_{-k+1}^{k},B_{-k}^{k}]$$

where the complex‐valued Wigner coefficients are defined as:

(C15)
$$\begin{array}{c} B_{q}^{k}=\left\{\begin{array}{lll} \left(B_{\left|q\right|}^{k}+\text{i}{B^{\prime }}_{\left|q\right|}^{k}\right) & \text{for} & q<0\\ B_{0}^{k} & \text{for} & q=0\\ {\left(-1\right)}^{\left|q\right|}(B_{\left|q\right|}^{k}-\text{i}{B^{\prime }}_{\left|q\right|}^{k}) & \text{for} & q>0 \end{array}\right. \end{array}$$

The rotation matrix is applied as follows:

(C16)
$$B^{\text{rot},k}=D^{k}B^{k}$$

where the elements of $D^{k}$ are organized according to the elements of $B^{k}$. The real‐valued rotated CFPs $B_{q}^{k}$ and ${B^{\prime }}_{q}^{k}$ are defined as real and imaginary part respectively of the q=−k,…,0 elements of $B^{\text{rot},k}$.
