# Supplementary material for: Not Just Another Crystal Field Software
Source: J Comput Chem. 2025 Mar 3;46(6):e70063. doi: 10.1002/jcc.70063 (PMC11875226; doi:10.1002/jcc.70063)
Supplement: Supplementary file 1 — Data S1. Additional supporting information may be found in the online version of the article at the publisher’s website. [file JCC-46-0-s001.pdf]

NJA-CFS: Not Just Another - Crystal Field Software

Letizia Fiorucci[1,2] and Enrico Ravera[1,2,3]

[1]CERM and Department of Chemistry "Ugo Schiff", University of Florence, Sesto Fiorentino, 50019, Italy
[2]Consorzio Interuniversitario Risonanze Magnetiche di Metalloproteine, Sesto Fiorentino, 50019, Italy
[3]Florence Data Science, University of Florence, Firenze, 50019, Italy

S1 Classification of states

As anticipated in the main text, the tensor operator representation of Hamiltonian contributions enables the application of the Wigner-Eckart theorem

$$\langle \alpha K M_K | T_q^{(k)} | \alpha' K' M_K' \rangle = (-1)^{K-M_K} \begin{pmatrix} K & k & K' \\ -M_K & q & M_K' \end{pmatrix} \langle \alpha K \| T^{(k)} \| \alpha' K' \rangle, \tag{S1}$$

where K and M_K are the angular momentum quantum numbers used to discriminate different states. The product of the first two terms on the r.h.s. is a vector coupling coefficient, while the remaining term is called reduced matrix element (RME).

For l^n with $n > 2$ the possibility of the occurrence of more than one term with the same L and S raises the problem of defining a state. So, a classification like the following should be used:

$$|l^n \alpha S L M_S M_L\rangle \tag{S2}$$

where α would be the additional quantum number used for terms' differentiation.

Considering that S, L, M_L, M_S are group-theoretical in origin, since e.g. the $2L+1$ components of a multiplet (with given S and M_S) transform under rotation according to the irreducible representation $\mathfrak{D}_L$ of R_3, the same principle could be used for other continuous groups that include the operations of R_3. The method for identifying such groups and for applying tensor operators to the theory of continuous groups are due to Racah[1].

Racah found that the groups that could serve for such scope are (in order): $R_3 \subset G_2 \subset R_7 \subset U_7$. The quantum numbers associated with each group are labels for their irreducible representations. G_2 is represented by $U = (u_1, u_2)$ and R_7 is represented by $W = (w_1, w_2, w_3)$. The representation of R_3 (and its subgroups) are the usual orbital angular momentum quantum numbers and the use of irreducible representations of U_7 (represented as $[\lambda_1, \lambda_2, ..., \lambda_n]$) is equivalent to the specification of the spin quantum number S. The decomposition schemes for the reductions $U_7 \to R_7$, $R_7 \to G_2$ and $G_2 \to R_3$ are reported in tables 5-1, 5-2 and 5-3 of [2] respectively.

However, in few cases, also this classification scheme is not enough, and Racah solved these few ambiguities arbitrarily in his work [3].

Additionally, Racah introduced the seniority quantum number[3], that is (for a certain S, W pair) represented by an integer ν such that f^ν is the first configuration in which that S, W appears.

This problem is not present for d^n configurations, whose state classification is straightforward. For this reason, in NJA-CFS the states are classified, following the example in f_electron

(available at the link: https://github.com/octoYot/f_electron), with the combination of 5 numbers for d^n configurations, i.e. S, L, J, M_J, ν, and 6 numbers for the f^n configurations, i.e. S, L, J, M_J, ν, γ, where the additional quantum number γ follows the Nielson e Koster classification and represents the unique combination of ν,U,W,S,L (plus the rare case where the arbitrary discrimination is needed).

The immediate advantage of the group theoretical classification of states is that many states can be uniquely defined without going through the lengths of calculating the particular linear combination of determinantal product states to which they correspond. To fully deploy the potential of this approach, it is necessary to find a different way to evaluate the matrix elements, not requiring the construction of such linear combinations. Racah[4] developed an elegant solution to this problem.

According to the definition in [2], assuming that Ω refers to the set of quantum numbers describing the state of l^n configuration, $\bar{\Omega}$ the set of quantum numbers for the state of l^{n-1} and ω the quantum numbers for the one-electron state, every determinantal product state for an n-electron system is a sum of terms

$$|\Omega\rangle = \sum_{\bar{\Omega},\omega} \langle\bar{\Omega};\omega|\Omega\rangle |\bar{\Omega}\rangle |\omega_n\rangle, \tag{S3}$$

where $\langle\bar{\Omega};\omega|\Omega\rangle$ are the coefficients of our interest and the subscript n indicates that the last state in the equation refers to the nth electron.

Using this equation, the matrix elements for a generic single-particle operator $F = \sum_i f_i$, between two states of the l^n configuration, can be expressed as:

$$\langle\Omega|F|\Omega'\rangle = n \sum_{\bar{\Omega},\omega,\omega'} \langle\Omega|\bar{\Omega};\omega\rangle \langle\omega_n|f_n|\omega_n\rangle \langle\bar{\Omega};\omega'|\Omega'\rangle \tag{S4}$$

while, for a two-particles operator $G = \sum_{i>j} g_{ij}$:

$$\langle\Omega|G|\Omega'\rangle = [n/(n-2)] \sum_{\bar{\Omega},\bar{\Omega}',\omega} \langle\Omega|\bar{\Omega};\omega\rangle \langle\bar{\Omega}| \sum_{j<i\neq n} g_{ij} |\bar{\Omega}'\rangle \langle\bar{\Omega}';\omega|\Omega'\rangle \tag{S5}$$

S2 From one-electron LF matrix elements to CFPs

Since, for NJA-CFS, we are interested in Wybourne coefficients expressed as a combination of two real numbers (B^k_q and B'^k_q), we implemented this procedure using the ligand-field matrix elements in the real basis that we can get from AILFT and the **C** matrix obtained exploiting the relations between the real basis and the complex basis:

$$|0\rangle = |\sigma\rangle, \tag{S6}$$

$$|1\rangle = -\frac{\mathrm{i}}{\sqrt{2}}|\pi_s\rangle - \frac{1}{\sqrt{2}}|\pi_c\rangle, \tag{S7}$$

$$|-1\rangle = -\frac{\mathrm{i}}{\sqrt{2}}|\pi_s\rangle + \frac{1}{\sqrt{2}}|\pi_c\rangle, \tag{S8}$$

$$|2\rangle = \frac{\mathrm{i}}{\sqrt{2}}|\delta_s\rangle + \frac{1}{\sqrt{2}}|\delta_c\rangle, \tag{S9}$$

$$|-2\rangle = -\frac{\mathrm{i}}{\sqrt{2}}|\delta_s\rangle + \frac{1}{\sqrt{2}}|\delta_c\rangle, \tag{S10}$$

$$|3\rangle = -\frac{\mathrm{i}}{\sqrt{2}}|\phi_s\rangle - \frac{1}{\sqrt{2}}|\phi_c\rangle, \tag{S11}$$

$$|-3\rangle = -\frac{\mathrm{i}}{\sqrt{2}}|\phi_s\rangle + \frac{1}{\sqrt{2}}|\phi_c\rangle, \tag{S12}$$

where the complex basis is indicated as $|l, m_l\rangle = |m_l\rangle$, while the real basis is indicated with $|l, u\rangle = |u\rangle$ (with $u = \sigma, \pi_s, \pi_c, \delta_s, \delta_c, \phi_s, \phi_c$). We chose this convention for the real orbitals since it is the one used in the papers on the subject[5]. The expressions are the same as the one reported in table 2 of [6] for d^n configurations and table 3 of [5] for f^n configurations.

The V^{LF} matrix elements can be computed ab initio through what is called: ab initio ligand field theory (AILFT)[7, 8] (see methods section and the section "Parameters from ab initio calculations" in the main text).

The one-electron ligand field matrices of AILFT from an ORCA CASSCF calculation (software v6.0) are organized as follows: for d^n configurations ($l = 2$), the elements are displayed, using the ORCA's orbital labels, as $[d_{z^2}, d_{xz}, d_{yz}, d_{x^2-y^2}, d_{xy}]$ (equivalent to $[\sigma, \pi_c, \pi_s, \delta_c, \delta_s]$), while for f^n configurations ($l = 3$), the elements are displayed as $[f_0, f_{+1}, f_{-1}, f_{+2}, f_{-2}, f_{+3}, f_{-3}]$ (equivalent to $[\sigma, \pi_c, \pi_s, \delta_c, \delta_s, \phi_c, \phi_s]$).

S3 NJA-CFS code listings and additional figures and tables

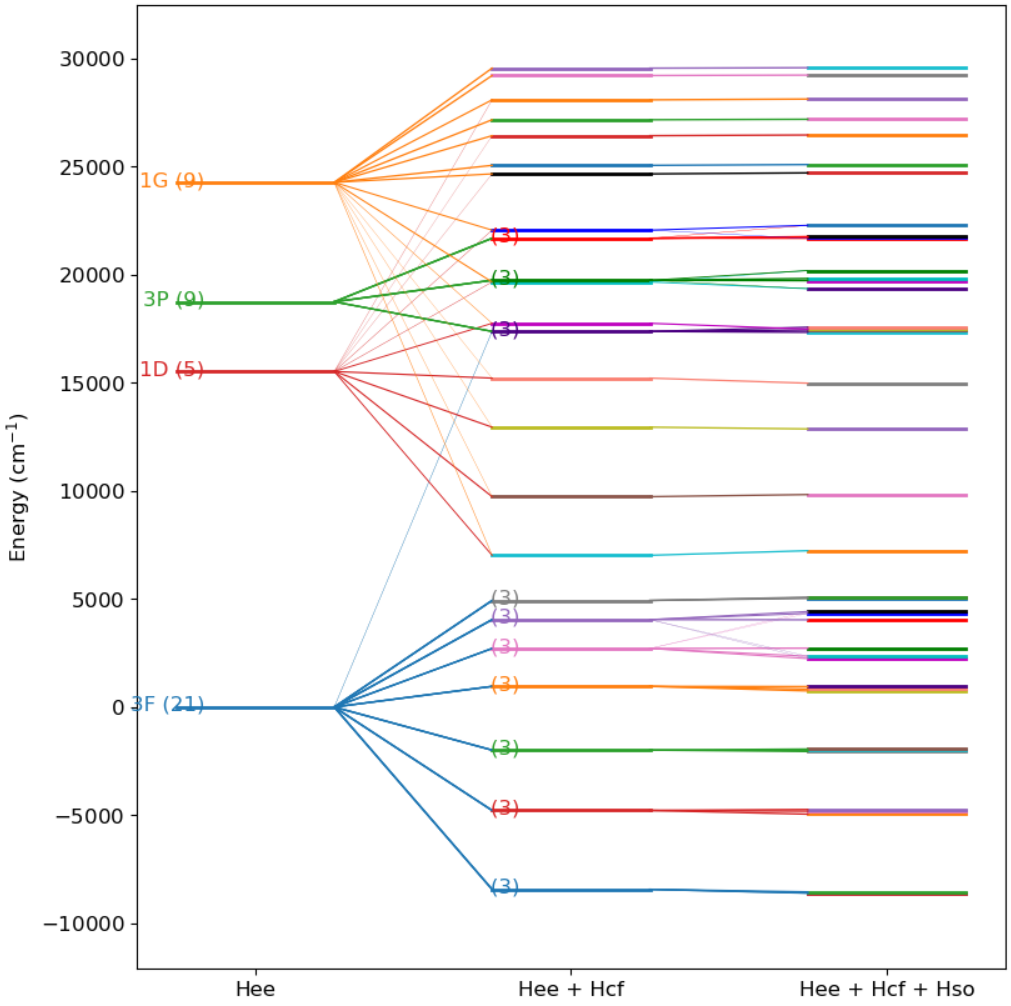

Figure S1: Energy levels splitting diagram for the $3d^8$ NiSAL-HDPT complex[9]. The parameters for the CF effective Hamiltonian come from a CASSCF(8,5) calculation performed with ORCA software (see Methods section in the main text). The free ion term state labels are also indicated, together with the $|LSJ\rangle$ degeneracy. The thickness of the lines connecting different levels is proportional to their relative contribution to the state composition. The energy of the ground-level free ion term is set to zero. The code listing for the calculation of this diagram is given in listing S2.

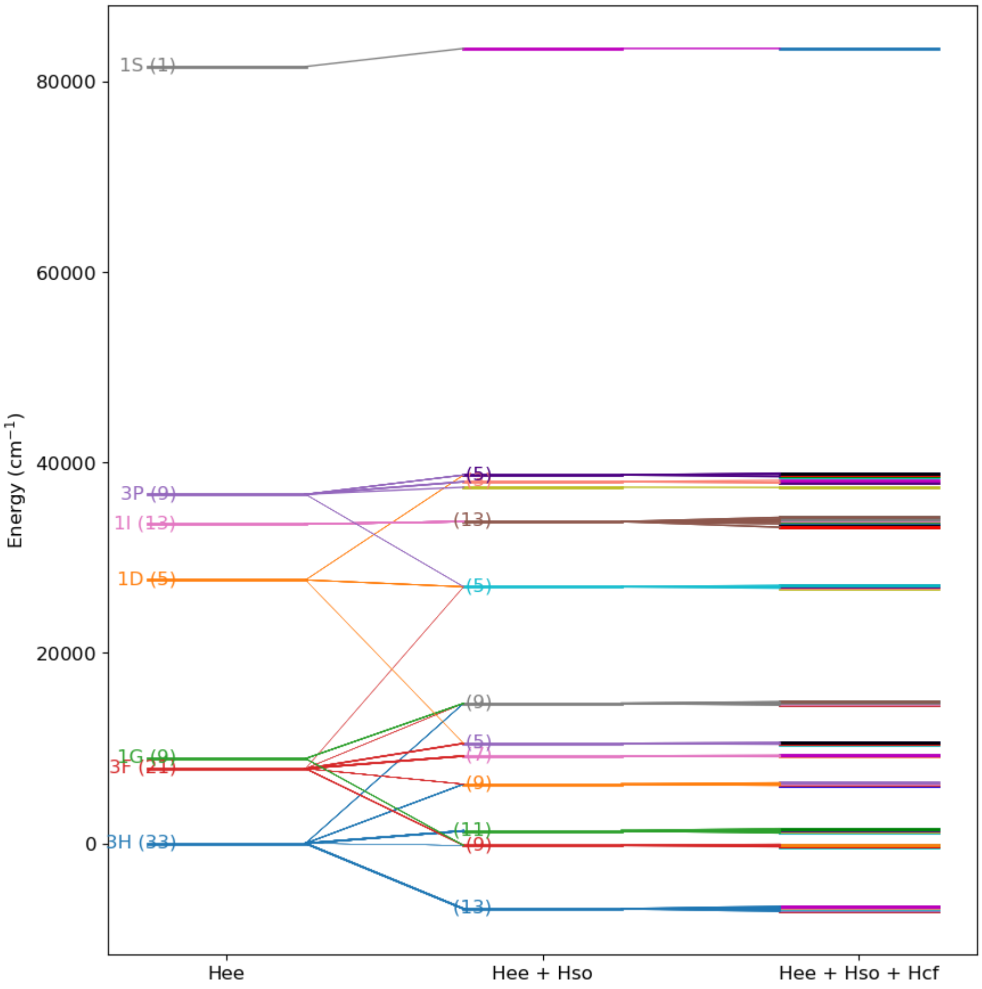

Figure S2: Energy levels splitting diagram for an $4f^{12}$ configuration in the PCM model of β-diketonate complex[10]. The Slater-Condon parameters and ζ were taken from [11], with $\kappa = 1$. The free ion term state label is also indicated, together with the $|LSJ\rangle$ degeneracy. The thickness of the lines connecting different levels is proportional to their relative contribution to the state composition. The energy of the ground-level free ion term is set to zero. The code listing for the calculation of this diagram is given in listing S3.

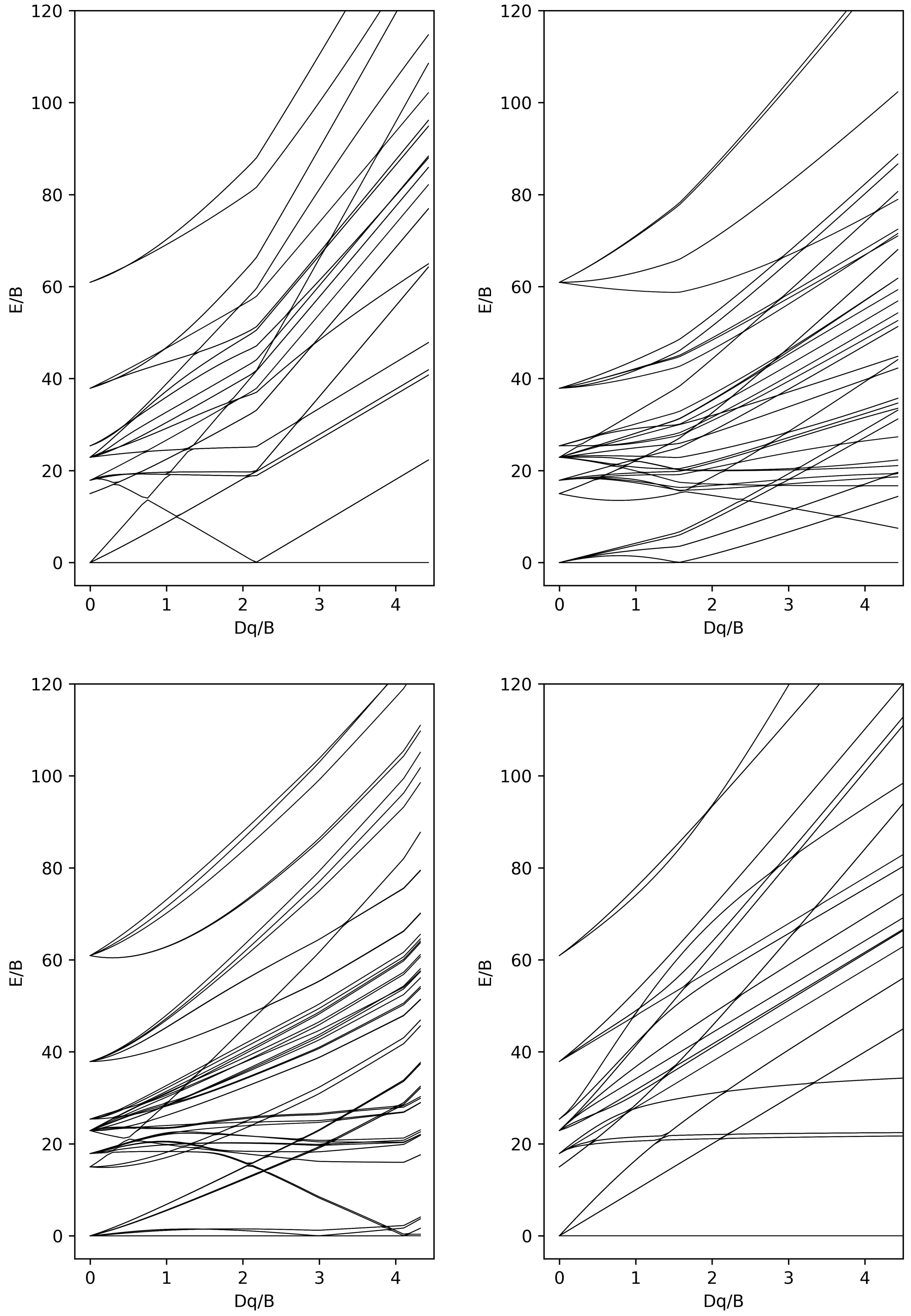

Figure S3: Tanabe-Sugano diagrams for O_h, C_{4v}, D_{3h} and T_d symmetries for $3d^7$ configuration, with Racah's parameter $B = 971$ cm^{-1}[12]. Only the electron-electron interaction and crystal field splitting were included in the CF effective Hamiltonian. The input files are reported in tables from S1 to S4 and the code in listing S4.

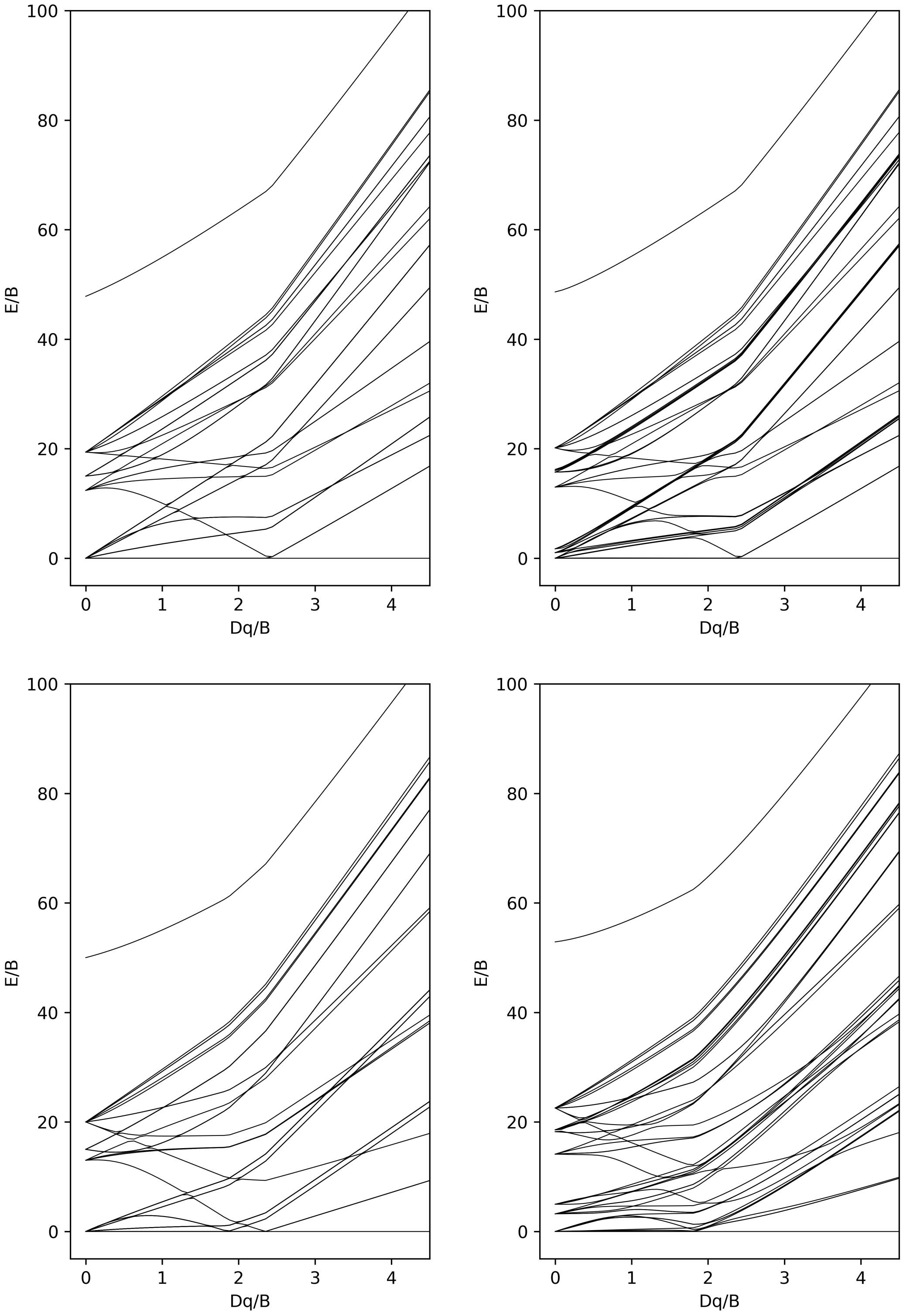
E/B
Dq/B

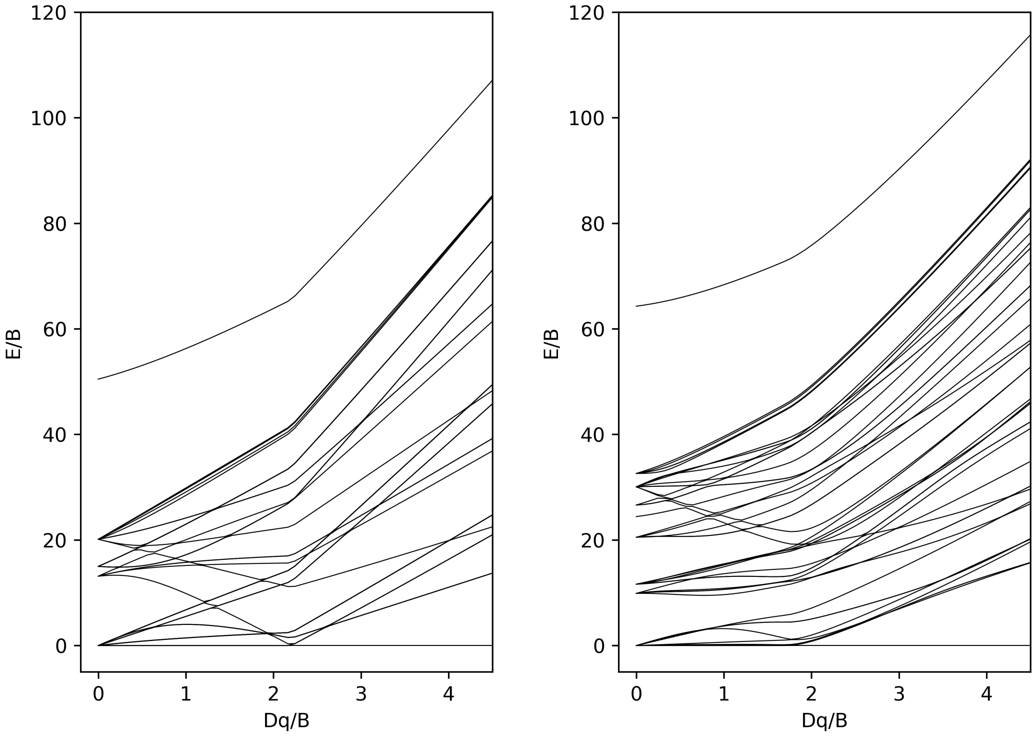

Figure S4: Tanabe-Sugano diagrams for D_{4h} symmetry in (starting from the top row) $3d^8$, $4d^8$ and $5d^8$ configurations. For each configuration, on the left is reported the diagram without SO coupling and on the right the same diagram obtained also considering the SO contribution in the Hamiltonian. The Racah's B values, F^k and ζ parameters were derived from CASSCF(8,5) calculations on PCMs of nickel(II), palladium(II) and platinum(II) respectively, as described in the Methods section of the main text. The B_q^k were computed from the same PCM and the charges and ligands positions were adjusted to get the same CF strength range for the entire series (analogously to what is done in listing S4).

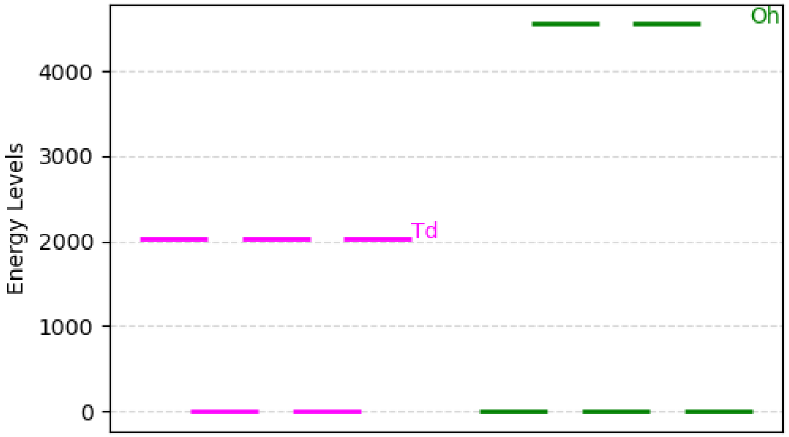

Figure S5: Orbital splitting diagram for O_h and T_d PCMs reported in tables S5 and S6, for a $3d^3$ configuration. The code is listed in S5.

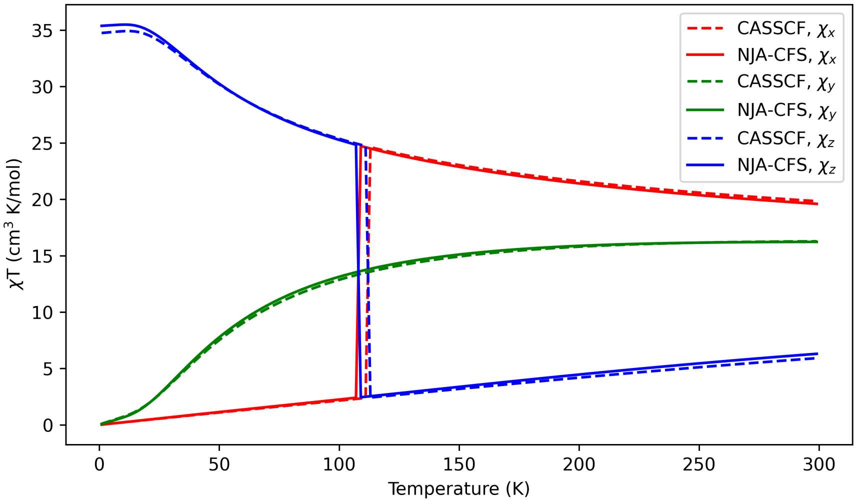

Figure S6: Temperature dependence of diagonal components of the magnetic susceptibility tensor for DyDOTA complex. The parameters used in NJA-CFS were obtained from the ab initio LF treatment implemented in ORCA, including all 21 sextuplets in the CASSCF(9,7) calculation. In the NJA-CFS simulation, the basis set was reduced to 21 sextuplets.

label	x (Å)	y (Å)	z (Å)	charge
Q_1	0.0	0.0	2.0	-2.1
Q_2	0.0	0.0	-2.0	-2.1
Q_3	2.0	0.0	0.0	-2.1
Q_4	-2.0	0.0	0.0	-2.1
Q_5	0.0	2.0	0.0	-2.1
Q_6	0.0	-2.0	0.0	-2.1

Table S1: PCM model for O_h symmetry assuming the metal center in $(0,0,0)$.

label	x (Å)	y (Å)	z (Å)	charge
Q_1	0.0	0.0	2.0	-1.4
Q_2	2.0	2.0	-1.414	-1.4
Q_3	-2.0	2.0	-1.414	-1.4
Q_4	-2.0	-2.0	-1.414	-1.4
Q_5	2.0	-2.0	-1.414	-1.4

Table S2: PCM model for C_{4v} symmetry assuming the metal center in $(0,0,0)$.

label	x (Å)	y (Å)	z (Å)	charge
Q_1	0.0	2.0	0.0	-0.9
Q_2	1.732	-1.091	0.0	-0.9
Q_3	-1.732	-1.091	0.0	-0.9

Table S3: PCM model for D_{3h} symmetry assuming the metal center in $(0,0,0)$.

label	x (Å)	y (Å)	z (Å)	charge
Q_1	1.633	0.0	-1.155	-2.0
Q_2	-1.633	0.0	-1.155	-2.0
Q_3	0.0	1.633	-1.155	-2.0
Q_4	0.0	-1.633	-1.155	-2.0

Table S4: PCM model for T_d symmetry assuming the metal center in $(0,0,0)$.

label	x (Å)	y (Å)	z (Å)	charge
Q_1	0.0	0.0	2.0	-1.0
Q_2	0.0	0.0	-2.0	-1.0
Q_3	2.0	0.0	0.0	-1.0
Q_4	-2.0	0.0	0.0	-1.0
Q_5	0.0	2.0	0.0	-1.0
Q_6	0.0	-2.0	0.0	-1.0

Table S5: PCM model for O_h symmetry assuming the metal center in $(0,0,0)$ constructed for the orbital splitting diagram in figure S5.

label	x (Å)	y (Å)	z (Å)	charge
Q_1	-1.1547	1.1547	1.1547	-1.0
Q_2	1.1547	-1.1547	1.1547	-1.0
Q_3	-1.1547	-1.1547	-1.1547	-1.0
Q_4	1.1547	1.1547	-1.1547	-1.0

Table S6: PCM model for T_d symmetry assuming the metal center in $(0,0,0)$ constructed for the orbital splitting diagram in figure S5.

label	x (Å)	y (Å)	z (Å)	charge
Cl	-0.219172	-2.70291	-0.040269	-1.0
O	2.037979	-0.038609	-0.6876060	-1.0
O	-1.86217	0.376778	0.935724	-1.0

Table S7: PCM model for Dybbpn complex[13]. The dysprosium(III) ion is assumed in (0,0,0).

label	x (Å)	y (Å)	z (Å)	charge
Cl	-2.72	0.0	0.0	-1.0
Cl	2.72	0.0	0.0	-1.0
Cl	0.0	-2.72	0.0	-1.0
Cl	0.0	2.72	0.0	-1.0
Cl	0.0	0.0	-2.72	-1.0
Cl	0.0	0.0	2.72	-1.0

Table S8: PCM model for $[DyCl_6]^{3-}$. The dysprosium(III) ion is assumed in (0,0,0).

label	x (Å)	y (Å)	z (Å)	charge
Q_1	-3.0	0.0	0.0	-1.0
Q_2	3.0	0.0	0.0	-1.0
Q_3	0.0	-3.0	0.0	-1.0
Q_4	0.0	3.0	0.0	-1.0

Table S9: Model complex used for constructing the Tanabe-Sugano diagrams in figure S4. The metal ions are assumed in (0,0,0).

Listing S1: Code listing for the comparison of optimized and not-optimized wavefunctions for CFPs computed in a PCM (see table S7) of the Dybbpn complex[13].

```
def eigenfunction_optimization_opt():

    #import NJA-CFS program and additional dependencies
    import nja_cfs as nja
    import numpy as np
    import matplotlib.pyplot as plt

    #choose the configuration
    conf = 'f9'

    #read the point charge model from file
    data = nja.read_data('test/bbpn.inp', sph_flag = False)
    data[:,-1] *= -1
```

```
    #compute the Bkq from the PCM
    dic_Bkq = nja.calc_Bkq(data, conf)

    #define the calculation object
    calc = nja.calculation(conf, ground_only=True, TAB=True, wordy=False
        )

    #define the parameters dictionary
    dic = {'dic_bkq': dic_Bkq}

    #compute the energy levels and wavefunctions by calling the MatrixH
        () routine (with eig_opt=True)
    result, projected = calc.MatrixH(['Hcf'], **dic, eig_opt=True, wordy
        =True, ground_proj=True, return_proj=True)

    plt.figure(figsize=(8, 6))
    plt.imshow(np.abs(result[1:,:]), cmap='viridis', interpolation='none
        ')
    plt.colorbar(label='Value')
    plt.xlabel('Column Index')
    plt.ylabel('Row Index')
    plt.xticks(ticks=np.arange(result.shape[1]), labels=np.arange(result
        .shape[1]))
    plt.yticks(ticks=np.arange(result.shape[1]), labels=np.arange(result
        .shape[1]))
    plt.show()

    dic = {'dic_bkq': dic_Bkq}
    #compute the energy levels and wavefunctions by calling the MatrixH
        () routine (with eig_opt=False)
    result, projected = calc.MatrixH(['Hcf'], **dic, eig_opt=False,
        wordy=True, ground_proj=True, return_proj=True)

    plt.figure(figsize=(8, 6))
    plt.imshow(np.abs(result[1:,:]), cmap='viridis', interpolation='none
        ')
    plt.colorbar(label='Value')
    plt.xlabel('Column Index')
    plt.ylabel('Row Index')
    plt.xticks(ticks=np.arange(result.shape[1]), labels=np.arange(result
        .shape[1]))
    plt.yticks(ticks=np.arange(result.shape[1]), labels=np.arange(result
        .shape[1]))
    plt.show()
```

Listing S2: Code listing for the calculation of energy levels splitting for the NiSAL complex (d^8 configuration), with LF effective hamiltonian parameters computed ab initio.

```
def test_plot_Ediagram():

    #import the NJA-CFS program and additional dependencies
    import nja_cfs as nja
    import numpy as np

    #choose the configuration
    conf = 'd8'
```

```
#initialize the calculation() object
calc = nja.calculation(conf, TAB=False, wordy=True)

#export the basis
basis, dic_LS, basis_l, basis_l_JM = nja.Full_basis(conf)

#read the parameters dictionary from an ORCA AILFT output file
dic_orca = nja.read_AILFT_orca6('test/orcaout_nisal.out', conf,
    method='CASSCF', return_V=False, rotangle_V=False,
    print_orcamatrix=False)

#define the Hamiltonian contributions and the splitting sequence
contributes = ['Hee', 'Hcf', 'Hso']
theories = ['Hee', 'Hee + Hcf', 'Hee + Hcf + Hso']

#collect the energy levels and projections for each "level of theory
    " defined in theories
list_contr = []
E_matrix = []
proj_LS_dict = {}
proj_prev_dict = {}
prev = np.zeros((basis.shape[0]+1,basis.shape[0]), dtype='complex128
    ')
for i in range(len(contributes)):
    list_contr.append(contributes[i])
    result = calc.MatrixH(list_contr, **dic_orca, field
        =[0.0,0.0,28.0], evaluation=True, wordy=True, ground_proj=
        False, return_proj=False)
    if i==0:
        E0 = np.min(result[0,:].real)
    result[0,:] = result[0,:].real-E0

    proj_LS = nja.projection_basis(result[1:,:], basis_l, J_label=
        False)

    proj_LS_dict[theories[i]] = proj_LS
    if i==0:
        pass
    else:
        proj_prev = nja.projection(result[1:,:], basis_l, prev
            [1:,:], prev[0,:].real)
        proj_prev_dict[theories[i]] = proj_prev

    E_matrix.append([round(result[0,ii].real,3) for ii in range(
        result.shape[-1])])  #tengo fino alla terza decimale

    prev = result.copy()

E_matrix = np.array(E_matrix)  # shape = (n. of contributes x n. of
    energy levels)

#plot of the energy levels splitting diagram
nja.fig_levels_splitting(E_matrix, theories, proj_LS_dict,
    proj_prev_dict)
```

Listing S3: Code listing for the calculation of energy levels splitting for a f^{12} configuration

in the β-diketonate PCM[10]. The Slater-Condon parameters and ζ are taken from tables in `free_ion_param_f_HF()`[11].

```
def test_plot_Ediagram():

    import nja_cfs as nja
    import numpy as np

    conf = 'f12'
    calc = nja.calculation(conf, TAB=False, wordy=True)
    basis, _, basis_l, _ = nja.Full_basis(conf)

    #compute the Bkq from the PCM using the calc_Bkq() function
    data = nja.read_data('test/beta.inp', sph_flag = False)
    data[:,-1] *= -1  #the multiplication by -1 expresses the charges as
         fractions of electronic charge
    dic_Bkq = nja.calc_Bkq(data, conf, False, True)
    dic_PCM = nja.free_ion_param_f_HF(conf)
    dic_PCM['dic_bkq'] = dic_Bkq

    contributes = ['Hee', 'Hso', 'Hcf']
    theories = ['Hee', 'Hee + Hso', 'Hee + Hso + Hcf']
    list_contr = []
    E_matrix = []
    proj_LS_dict = {}
    proj_prev_dict = {}
    prev = np.zeros((basis.shape[0]+1,basis.shape[0]), dtype='complex128
        ')
    for i in range(len(contributes)):
        list_contr.append(contributes[i])
        result = calc.MatrixH(list_contr, **dic_PCM, field
            =[0.0,0.0,28.0], evaluation=True, wordy=True, ground_proj=
            False, return_proj=False)
        if i==0:
            E0 = np.min(result[0,:].real)
        result[0,:] = result[0,:].real-E0

        proj_LS = nja.projection_basis(result[1:,:], basis_l, J_label=
            False)

        proj_LS_dict[theories[i]] = proj_LS
        if i==0:
            pass
        else:
            proj_prev = nja.projection(result[1:,:], basis_l, prev
                [1:,:], prev[0,:].real)
            proj_prev_dict[theories[i]] = proj_prev

        E_matrix.append([round(result[0,ii].real,3) for ii in range(
            result.shape[-1])])

        prev = result.copy()

    E_matrix = np.array(E_matrix)

    nja.level_fig_tot(E_matrix, theories, proj_LS_dict, proj_prev_dict)
```

Listing S4: Code listing for the generation of Tanabe-Sugano plots for a $3d^7$ configuration.

```
def test_TanabeSugano():

    import nja_cfs as nja
    import numpy as np
    import matplotlib.pyplot as plt

    conf = 'd7'
    B = 971

    data = nja.read_data('test/<inputfilename>.inp', sph_flag = False)
    data[:,-1] *= -1

    calc = nja.calculation(conf, ground_only=False, TAB=True, wordy=
        False)

    #first point at 0 CF
    dic = nja.free_ion_param_AB(conf)
    result = calc.MatrixH(['Hee'], **dic, eig_opt=False, wordy=False)
    proj_LS = nja.projection_basis(result[1:,:], calc.basis_l)

    diagram = [(result[0,:]-np.min(result[0,:]))/B]
    x_axis = [0.0]
    spacing = np.arange(0.01,12,0.2)
    print(len(spacing))
    for i in range(len(spacing)):
        data_mult = copy.deepcopy(data)
        data_mult[:,-1] *= spacing[i]
        dic = nja.free_ion_param_AB(conf)
        dic_Bkq = nja.calc_Bkq(data_mult, conf, False, False)
        dic_V = nja.from_Vint_to_Bkq_2(2, dic_Bkq, reverse=True)
        matrix = np.zeros((5,5))
        for ii in range(5):
            for j in range(5):
                if ii>=j:
                    matrix[ii,j] = dic_V[str(ii+1)+str(j+1)]
                    matrix[j,ii] = dic_V[str(ii+1)+str(j+1)]
        w,v = np.linalg.eigh(matrix)
        x_axis.append(-(w[0]-w[-1])/B)
        dic['dic_bkq'] = dic_Bkq
        result = calc.MatrixH(['Hee','Hcf'], **dic, eig_opt=False, wordy
            =False)
        diagram.append((result[0,:]-np.min(result[0,:]))/B)

    diagram = np.array(diagram)
    fig, ax = plt.subplots()
    for i in range(diagram.shape[1]):
        ax.plot(np.array(x_axis)/10, diagram[:,i].real, 'k', lw=0.5)
    plt.show()
```

Listing S5: Code listing for the calculation of the orbital splitting diagrams for O_h and T_d symmetries, for a complex in $3d^3$ configuration.

```
def test_CF_splitting():

    import nja_cfs as nja
```

```
import numpy as np
import matplotlib.pyplot as plt

# Example usage to add multiple crystal field splittings
fig, ax = plt.subplots()

conf = 'd3'
contributes = ['Hee', 'Hcf', 'Hso']

data = nja.read_data('test/Td_cube.inp', sph_flag = False)
data[:,-1] *= -1
data[:,1:-1] *= 2/(2*np.sqrt(3))

dic_Bkq = nja.calc_Bkq(data, conf, False, False)
dic_V = nja.from_Vint_to_Bkq_2(2, dic_Bkq, reverse=True)
matrix = np.zeros((5,5))
for i in range(5):
    for j in range(5):
        if i>=j:
            matrix[i,j] = dic_V[str(i+1)+str(j+1)]
            matrix[j,i] = dic_V[str(i+1)+str(j+1)]

w,v = np.linalg.eigh(matrix)

nja.plot_energy_levels(w-np.min(w), ax=ax, color='magenta', label="
    Td", delta=0)

data = nja.read_data('test/Oh_cube.inp', sph_flag = False)
data[:,-1] *= -1
dic_Bkq = nja.calc_Bkq(data, conf, False, False)
dic_V = nja.from_Vint_to_Bkq_2(2, dic_Bkq, reverse=True)
matrix = np.zeros((5,5))
for i in range(5):
    for j in range(5):
        if i>=j:
            matrix[i,j] = dic_V[str(i+1)+str(j+1)]
            matrix[j,i] = dic_V[str(i+1)+str(j+1)]

w,v = np.linalg.eigh(matrix)

nja.plot_energy_levels(w-np.min(w), ax=ax, color='green', label="Oh"
    , delta=0.5)

plt.show()
```

Listing S6: This function computes the numerical derivative of a function (`func`) with respect to the variable `x`, with derivation step of `h`. It returns both the value of the derivative and the error estimation. The maximum number of `func` evaluations can be as large as $2 \times$ `NTAB`. The value of `h`, which is the estimated initial stepsize, has to be large enough to produce a sensible variation in the `func` evaluation. This code was adapted from the original Fortran implementation of Ridders' method of polynomial extrapolation available in [14]. This routine is applied in NJA-CFS for the computation of the susceptibility tensor and to estimate the error from the numerical derivative.

```
def dfridr(func, x, h, idxi, shape, fargs):
```

```
    CON = h*2
    CON2 = CON * CON
    NTAB = 10
    SAFE = 2
    a = np.zeros((NTAB, NTAB)+shape[1:])

    hh = h
    zero = 1e-16

    dx = np.copy(x)
    dx[idxi] += hh
    sx = np.copy(x)
    sx[idxi] -= hh
    if 2*hh!=0:
        a[0,0,...] = ((func(dx,*fargs)-func(sx,*fargs))/(2*hh))
    else:
        a[0,0,...] = ((func(dx,*fargs)-func(sx,*fargs))/zero)

    err = np.Inf
    result = None

    for i in range(1, NTAB):
        hh /= CON
        dx = np.copy(x)
        dx[idxi] += hh
        sx = np.copy(x)
        sx[idxi] -= hh
        if 2*hh!=0:
            a[0,i,...] = ((func(dx,*fargs)-func(sx,*fargs))/(2*hh))
        else:
            a[0,i,...] = ((func(dx,*fargs)-func(sx,*fargs))/zero)
        fac = CON2
        for j in range(1, i):
            if (fac - 1)!=0:
                a[j, i,...] = (a[j - 1, i,...] * fac - a[j - 1, i -
                    1,...]) / (fac - 1)
            else:
                a[j, i,...] = (a[j - 1, i,...] * fac - a[j - 1, i -
                    1,...]) / zero
            fac *= CON2
            errt = max(norm(a[j, i,...] - a[j - 1, i,...]), norm(a[j, i
                ,...] - a[j - 1, i - 1,...]))
            if errt <= err:
                err = errt
                result = a[j, i,...]
        if norm(a[i, i,...] - a[i - 1, i - 1,...])
        >= SAFE * err:
            return result, err

    return result, err
```

Listing S7: Computation and plot of magnetic susceptibility tensor for the DyDOTA with B_k^q taken from [15]

```
def test_susceptibility_B_ord1_3():

    import nja_cfs as nja
```

```
    import numpy as np

    conf = 'f9'
    contributes = ['Hcf']

    cfp_list = np.loadtxt('test/CFP_DyDOTA.txt')
    dic_Aqkrk = {}
    count = 0
    for k in range(2,7,2):
        dic_Aqkrk[f'{k}'] = {}
        for q in range(k,-k-1,-1):
            dic_Aqkrk[f'{k}'][f'{q}'] = cfp_list[count]/nja.Stev_coeff(
                str(k), conf)
            count += 1

    dic_Bkq = nja.from_Aqkrk_to_Bkq(dic_Aqkrk)
    dic = {}
    dic['dic_bkq'] = dic_Bkq
    dic_Bkq['0'] = {}
    dic_Bkq['0']['0'] = 0

    Rot_mat = np.array([[0.696343, 0.027550, -0.717180],[0.216884,
        0.944468, 0.246864],[0.684155, -0.327447, 0.651698]])

    R = scipy.spatial.transform.Rotation.from_matrix(Rot_mat.T).as_quat
        ()
    quat = [R[-1], R[0], R[1], R[2]]
    dict, coeff = nja.read_DWigner_quat()
    dic_Bkq = nja.rota_LF_quat(3, dic_Bkq, quat, dict=dict, coeff=coeff)
    dic['dic_bkq'] = dic_Bkq
    dic_Bkq['0'] = {}
    dic_Bkq['0']['0'] = 0

    calc = nja.calculation(conf, ground_only=True, TAB=True, wordy=False
        )
    _, _ = calc.MatrixH(contributes, **dic, eig_opt=False, wordy=False,
        ground_proj=True, return_proj=True, save_label=True, save_LF=
        True)
    basis = np.loadtxt('matrix_label.txt')
    LF_matrix = np.load('matrix_LF.npy', allow_pickle=True, fix_imports=
        False)

    chi_B_diff, err_B = nja.susceptibility_B_ord1(np.array
        ([[0.0,0.0,0.0]]), 2., basis, LF_matrix, delta=1)

    w,v = np.linalg.eig(chi_B_diff)

    nja.fig_tensor_rep_1(chi_B_diff)
```

Listing S8: Code listing for the calculation of the magnetic torque of a mole of $TbPc_2$ for three different fields at 2 K. The field is placed along z and the plotted component of the torque is along y. The $A_k^q\langle r^k\rangle$ are taken from [16].

```
def test_torque():

    import nja_cfs as nja
    import numpy as np
```

```
    conf = 'f8'
    contributes = ['Hcf']

    dic_Aqkrk = {'2':{'0':293.0},
                 '4':{'0':-197.0, '4':863.0},
                 '6':{'0':15.1, '4':357.0}}

    dic_Bkq = nja.from_Aqkrk_to_Bkq(dic_Aqkrk)
    dic = {}
    dic['dic_bkq'] = dic_Bkq

    calc = nja.calculation(conf, ground_only=True, TAB=True, wordy=False
        )
    _, _ = calc.MatrixH(contributes, **dic, eig_opt=False, wordy=False,
        ground_proj=True, return_proj=True, save_label=True, save_LF=
        True)
    basis = np.loadtxt('matrix_label.txt')
    LF_matrix = np.load('matrix_LF.npy', allow_pickle=True, fix_imports=
        False)

    B0 = [0.1, 2, 5] #T
    T = 2.0 #K
    #returns angles and torque values
    #if plane is 'zx' then the returned tau is along the y axis and the
        field is placed on z (i.e. the first axis in 'zx' label)
    x,y = nja.calc_torque(B0, T, LF_matrix, basis, plane='zx', figure='
        figurename.png', show_fig=True)
```

Listing S9: Code listing for the reduction of the basis set used for the computation of the energy levels for a dysprosium(III) complex.

```
def test_reduction():

    import nja_cfs as nja

    conf = 'f9'
    contributes = ['Hee','Hso','Hcf']
    dic = nja.read_AILFT_orca6('test/run_DOTA1_21sextets.out', conf)

    calc = nja.calculation(conf, ground_only=False, TAB=False, wordy=
        True)
    calc.reduce_basis(conf, roots = [(21,6)], wordy=True)
    result, projected = calc.MatrixH(contributes, **dic, eig_opt=False,
        wordy=True, ground_proj=True, return_proj=True)
```

Listing S10: Code listing for the computation of the susceptibility temperature dependence of $Cs_2NaDyCl_6$. The reader should refer to figure 7 and table 2 of the main text.

```
def comparison_exp_chi():

    import nja_cfs as nja
    import numpy as np
    import matplotlib.pyplot as plt
```

```
## experimental data
x = np.array([2.842, 4.918, 5.027, 6.230, 7.322, 8.634, 9.945,
    11.257, 12.678, 14.863, 16.175, 17.596, 19.126, 20.874, 23.934,
    27.104, 28.525, 30.164, 31.585, 33.333, 36.503, 39.344, 41.421,
    44.372, 47.432, 50.601, 53.661, 56.612, 59.781, 62.514, 65.683,
    68.852, 71.913, 75.082, 78.251, 81.311, 84.481, 86.776])
y = np.array([6.300, 7.996, 7.996, 7.863, 8.524, 9.383, 9.581,
    9.978, 10.441, 11.806, 11.674, 12.115, 11.872, 12.533, 12.753,
    12.907, 13.018, 13.106, 13.172, 13.238, 13.392, 13.326, 13.502,
    13.414, 13.392, 13.546, 13.502, 13.524, 13.612, 13.590, 13.656,
    13.546, 13.546, 13.546, 13.502, 13.590, 13.612, 13.436])

conf = 'f9'

calc = nja.calculation(conf, ground_only=False, TAB=True, wordy=
    False)
calc.reduce_basis(conf, roots = [(21,6)], wordy=True)

## AILFT
dic = {}
dic_V = {
     '11':12140.0,
     '21':0, '22':11971.4,
     '31':0,'32':0,'33':11971.1,
     '41':0,'42':0,'43':0,'44':11735.3,
     '51':0,'52':0,'53':0,'54':0,'55':11870.1,
     '61':0,'62':130.7,'63':0,'64':0,'65':0,'66':12038.3,
     '71':0,'72':0,'73':-130.6,'74':0,'75':0,'76':0,'77':12038.9
}
dic_Bkq = nja.from_Vint_to_Bkq(dic_V, conf)
dic['dic_bkq'] = dic_Bkq
dic['F2'] = 109890.1
dic['zeta'] = 1737.8
result, projected = calc.MatrixH(['Hee','Hso','Hcf'], **dic, eig_opt
    =False, wordy=True, ground_proj=True, return_proj=True)
Magn = nja.Magnetics(calc, ['Hee','Hso','Hcf','Hz'], dic)
chi_nja = []
x1 = np.arange(1,90,4)
for T in x1:
     print(str(T), end='\r')
     chi= Magn.susceptibility_field(np.array([[0.0,0.0,1e-7]]), T,
         delta=0.01)[1]
     chi_nja.append(chi[0])
chi_nja = np.array(chi_nja)

## PCM dycl6^-3
data = nja.read_data('test/dycl63-.inp', sph_flag = False)
data[:,-1] *= -1
dic_Bkq = nja.calc_Bkq(data, conf)
dic['dic_bkq'] = dic_Bkq
result, projected = calc.MatrixH(['Hee','Hso','Hcf'], **dic, eig_opt
    =False, wordy=True, ground_proj=True, return_proj=True)
Magn = nja.Magnetics(calc, ['Hee','Hso','Hcf','Hz'], dic)
chi_nja2 = []
x1 = np.arange(1,90,4)
for T in x1:
     print(str(T), end='\r')
     chi= Magn.susceptibility_field(np.array([[0.0,0.0,1e-7]]), T,
         delta=0.01)[1]
```

```
    chi_nja2.append(chi[0])
chi_nja2 = np.array(chi_nja2)

## PCM cs2nadycl6
data = nja.read_data('test/cs2nadycl6.inp', sph_flag = False)
data[:,-1] *= -1
dic_Bkq = nja.calc_Bkq(data, conf)
dic['dic_bkq'] = dic_Bkq
result, projected = calc.MatrixH(['Hee','Hso','Hcf'], **dic, eig_opt
    =False, wordy=True, ground_proj=True, return_proj=True)
Magn = nja.Magnetics(calc, ['Hee','Hso','Hcf','Hz'], dic)
chi_nja4 = []
x1 = np.arange(1,90,4)
for T in x1:
    print(str(T), end='\r')
    chi= Magn.susceptibility_field(np.array([[0.0,0.0,1e-7]]), T,
        delta=0.01)[1]
    chi_nja4.append(chi[0])
chi_nja4 = np.array(chi_nja4)

## AILFT corrected for PCM cs2nadycl6
data = nja.read_data('test/cs2nadycl6_empty.inp', sph_flag = False)
data[:,-1] *= -1
dic_Bkq = nja.calc_Bkq(data, conf)
dic_V2 = nja.from_Vint_to_Bkq_2(3, dic_Bkq, reverse=True)
dic_V3 = {key:dic_V2[key]+dic_V[key] for key in dic_V2.keys()}
dic_Bkq = nja.from_Vint_to_Bkq_2(3, dic_V3)
dic['dic_bkq'] = dic_Bkq
result, projected = calc.MatrixH(['Hee','Hso','Hcf'], **dic, eig_opt
    =False, wordy=True, ground_proj=True, return_proj=True)
Magn = nja.Magnetics(calc, ['Hee','Hso','Hcf','Hz'], dic)
chi_nja5 = []
x1 = np.arange(1,90,4)
for T in x1:
    print(str(T), end='\r')
    chi= Magn.susceptibility_field(np.array([[0.0,0.0,1e-7]]), T,
        delta=0.01)[1]
    chi_nja5.append(chi[0])
chi_nja5 = np.array(chi_nja5)

## AOM dycl6^-3
data = nja.read_data('test/dycl63-.inp', sph_flag = False)
data[:,-1] *= -1
dic_Bkq = nja.calc_Bkq(data, conf)
del dic['dic_bkq']
AOM = np.array([[300.5,138,138,0.0,0.0,0.0],
                        [300.5,138,138,0.0,0.0,0.0],
                        [300.5,138,138,0.0,0.0,0.0],
                        [300.5,138,138,0.0,0.0,0.0],
                        [300.5,138,138,0.0,0.0,0.0],
                        [300.5,138,138,0.0,0.0,0.0]])
sph_coord = nja.from_car_to_sph(data[:,1:-1])
AOM[:,3:-1] = sph_coord[:,1:]*180/np.pi
dic_AOM = {}
for i in range(6):
    dic_AOM['Cl'+str(i+1)] = AOM[i,:]
dic['dic_AOM'] = dic_AOM
dic['F2'] = 412.1*225
dic['F4'] = 60.9*1089
```

```
dic['F6'] = 6.3*184041/25
dic['zeta'] = 1920
calc = nja.calculation(conf, ground_only=False, TAB=True, wordy=
    False)
calc.reduce_basis(conf, roots = [(21,6),(13,4)], wordy=True)
result, projected = calc.MatrixH(['Hee','Hso','Hcf'], **dic, eig_opt
    =False, wordy=True, ground_proj=True, return_proj=True)
Magn = nja.Magnetics(calc, ['Hee','Hso','Hcf','Hz'], dic)
chi_nja3 = []
x1 = np.arange(1,90,4)
for T in x1:
    print(str(T), end='\r')
    chi= Magn.susceptibility_field(np.array([[0.0,0.0,1e-7]]), T,
        delta=0.01)[1]
    chi_nja3.append(chi[0])
chi_nja3 = np.array(chi_nja3)

## plot of the results
conv = 1/(np.pi*4/(1e6*scipy.constants.Avogadro*x1)) #cm^3 K/mol
plt.figure(figsize=(5, 4.5))
plt.plot(x,y,'o',c='b',label='Experiment')
plt.ylim(0,15)
plt.plot(x1,chi_nja*conv,'-.',c='r',label='NJA-CFS (AILFT)')
plt.plot(x1,chi_nja2*conv,'--',c='r',label=r'NJA-CFS (PCM [DyCl$_6$]
    $^{-3}$)')
plt.plot(x1,chi_nja4*conv,'--',c='g',label=r'NJA-CFS (PCM
    Cs$_2$NaDyCl$_6$)')
plt.plot(x1,chi_nja5*conv,'-.',c='g',label='NJA-CFS (AILFT corrected
    )')
plt.plot(x1,chi_nja3*conv,':',c='magenta',label='NJA-CFS (AOM)')
plt.xlabel('Temperature (K)')
plt.ylabel(r'$\chi$T (cm$^3$ K/mol)')
plt.legend()
plt.show()
```

References

[1] Giulio Racah. *Group theory and spectroscopy*. Institute for Advanced Study, 1951.

[2] Brian R Judd. *Operator techniques in atomic spectroscopy*. Vol. 35. Princeton University Press, 2014.

[3] Giulio Racah. “Theory of complex spectra. IV”. In: *Physical Review* 76.9 (1949), p. 1352.

[4] Giulio Racah. “Theory of complex spectra. III”. In: *Physical Review* 63.9-10 (1943), p. 367.

[5] W Urland. “On the ligand-field potential for f electrons in the angular overlap model”. In: *Chemical Physics* 14.3 (1976), pp. 393–401.

[6] Malcolm Gerloch and Robert F McMeeking. “Paramagnetic properties of unsymmetrical transition-metal complexes”. In: *Journal of the Chemical Society, Dalton Transactions* 22 (1975), pp. 2443–2451.

[7] Mihail Atanasov et al. “A modern first-principles view on ligand field theory through the eyes of correlated multireference wavefunctions”. In: *Molecular electronic structures of transition metal complexes II* (2012), pp. 149–220.

[8] Lucas Lang, Mihail Atanasov, and Frank Neese. “Improvement of ab initio ligand field theory by means of multistate perturbation theory”. In: *The Journal of Physical Chemistry A* 124.5 (2020), pp. 1025–1037.

[9] Enrico Ravera et al. "A quantum chemistry view on two archetypical paramagnetic pentacoordinate nickel (II) complexes offers a fresh look on their NMR spectra". In: *Inorganic Chemistry* 60.3 (2021), pp. 2068–2075.

[10] Shang-Da Jiang and Si-Xue Qin. "Prediction of the quantized axis of rare-earth ions: the electrostatic model with displaced point charges". In: *Inorganic Chemistry Frontiers* 2.7 (2015), pp. 613–619.

[11] C-G Ma et al. "Systematic analysis of spectroscopic characteristics of the lanthanide and actinide ions with the 4fN and 5fN (N= 1... 14) electronic configurations in a free state". In: *Journal of alloys and compounds* 599 (2014), pp. 93–101.

[12] Anatole Abragam and Brebis Bleaney. *Electron paramagnetic resonance of transition ions*. OUP Oxford, 2012.

[13] Francielli S Santana et al. "A dysprosium single molecule magnet outperforming current pseudocontact shift agents". In: *Chemical Science* 13.20 (2022), pp. 5860–5871.

[14] William H Press et al. *Numerical recipes*. Cambridge University Press, London, England, 1988.

[15] Matteo Briganti et al. "Magnetic anisotropy trends along a full 4f-series: the fn+ 7 effect". In: *Journal of the American Chemical Society* 143.21 (2021), pp. 8108–8115.

[16] Naoto Ishikawa, Tomochika Iino, and Youkoh Kaizu. "Determination of ligand-field parameters and f-electronic structures of hetero-dinuclear phthalocyanine complexes with a diamagnetic yttrium (III) and a paramagnetic trivalent lanthanide ion". In: *The Journal of Physical Chemistry A* 106.41 (2002), pp. 9543–9550.
